# Measurement reliability, construct validity, and transparent reporting in original and replication psychological research

**DOI:** 10.1371/journal.pone.0356070

**Published:** 2026-09-02

**Authors:** Cas Goos, Marjan Bakker, Jelte M. Wicherts, Michèle B. Nuijten

**Affiliations:** Department of Methodology and Statistics, School of Social and Behavioral Sciences, Tilburg University, Tilburg, Netherlands; Fundación Universitaria del Área Andina, COLOMBIA

## Abstract

Published (replication) studies that use measures to assess psychological phenomena need to transparently report information on measurement procedures, validity, and reliability to allow verification and use in future (replication) research. However, earlier results highlighted widespread poor reporting of psychological measurement. Here, we investigated measurement reporting in a sample of 77 measures within 56 Many Labs replications and related original articles (14–17) and found that the information relevant for reusing measures was reported in full in around half the replication measures, and only in 5.2% of the original studies. We also observed that around a third of multiple-item measures in original studies and 11.4% in replications reported reliability coefficients, with comparable proportions for reporting any convergent, discriminant, predictive, or factorial validity evidence. We assessed the reliability, unidimensionality, and measurement invariance of multiple-item measures using the openly available Many Labs item response data. We observed that while some measures passed these psychometric checks, they rarely did so consistently across labs. These results corroborate existing findings that measurement reporting in published research lacks transparency, and that poor measurement reporting may obscure insufficient reliability and validity. We offer suggestions on how to improve measurement reporting practices and increase the use of validated measures.

## Introduction

Because psychological constructs cannot be directly measured, quantitative researchers use scores from measures to study psychological phenomena. Psychological measurement is no easy feat, and as a result, measures will not always form good indicators of the constructs they purport to measure. Measures need to be validated and associated with sufficient reliability to deal with systematic and unsystematic measurement errors so they can attribute observed variation in scores to the variance in the unobserved construct(s) of interest. Psychometrics offers a wide range of tools to assess reliability and to study validity. These range from simple measures of internal consistency like Cronbach’s alpha that can easily be calculated for any set of numeric data, to item response theory and structural equation models where the observed items scores are statistically modelled unto supposed latent variables, after which the fit of this model of latent variables can be tested.

In practice, however, when the reliability of psychological measures is recalculated they often exhibit subpar reliability. Furthermore, they are typically validated and psychometrically tested in limited circumstances, samples, and measurement procedures, while it is likely that measures do not behave the same across all relevant samples, locations, and time points [1]. Moreover, for many measures used in psychological research the reliability and construct validity remain unclear, either because the evidence is not reported in studies using them, or because the measures are not validated to begin with [2–6]. Such practices are dubbed “Questionable Measurement Practices” (QMPs; [7]), being a term analogous to Questionable Research Practices (QRPs; [8–10]). A recent line of research has already shown QMPs to be common in both original and replication psychology studies, damaging the trustworthiness and applicability of psychological research findings [6,11].

The purpose of this study is to assess the measurement reporting practices of item-based measures in a diverse sample of seminal original and replication studies in psychology. In addition, we will use the available item response data to calculate reliability indices and check for unidimensionality — as a prerequisite for establishing construct validity. By assessing the reported measurement information together with reliability and unidimensionality as calculated from the primary data, we reflect on the apparent validity of the measurement based on the reported information and novel analyses of openly shared item response data.

### Measurement reporting

Reported information on measurement (or its validity) is typically insufficient for readers to understand, evaluate, and reuse the used measurements. Flake et al. [6] documented measurement reporting practices among 100 original psychology articles and their respective replications from the Reproducibility Project Psychology [12]. They coded the number the content, reported information, and justification of the measures, and coded whether Cronbach’s alpha coefficients were reported. For validity evidence, they looked at citations to existing validity evidence from previous studies documenting the scale’s development or use, and results from a factor analysis providing structural evidence for the measure. They observed limited reporting of both reliability and validity evidence. Additionally, only 8 of the 40 translated scales referenced an existing validity study that described evidence for the translated version. A similar lack of reliability and validity reporting has been observed in other studies as well [4,13]. If articles fail to report evidence on to the reliability or validity evidence, then readers lack the basic information needed to assess whether the measured variables relate to the constructs of interest. In turn, if the reported information does not exhibit some minimal level of validity — through reliability coefficients, an existing validation study, or factor analysis results — then the substantive conclusions drawn from the data are left unsupported.

Further findings by Flake et al. [6] and others [4,5] show that issues in measurement reporting go beyond a lack of reporting reliability and validity evidence. Basic content descriptions such as the number of items, the response format, and the scoring of the scale are not always clearly reported. This creates challenges for future researchers who want to reuse the measure to assess the same construct. Specifically, replication studies attempting to reconstruct the measurement from these incomplete descriptions may end up with a measurement that assesses the constructs in a substantially different way from the original study or even assess different constructs altogether. If different constructs are assessed, the replication cannot be seen as a test of the same phenomenon as in the original study. If the same constructs are assessed, but in a substantially different way, the estimated effects in the replication cannot be easily compared to the effects in the original study. In either case, substantive comparisons between original and replication are hindered. As a result, critically testing theories is hindered because we cannot establish that the relevant constructs were assessed [14–16].

Our first aim is to extend the existing findings on measurement reporting practices with a descriptive account of these practices for the Many Labs replications and the related original studies. We will evaluate to what extent the reporting of item-based scales in our sample is transparent enough to facilitate the evaluation and reuse of these measurements. The Many Labs replications are a series of large-scale collaborations, in which multiple labs across the world directly replicated classic and contemporary psychological studies [17–21]. The original studies chosen for replication in the Many Labs were not only picked based on feasibility, but — importantly for us — the sample of effects to replicate was chosen to contain a diverse range of seminal effects. Furthermore, because the Many Labs projects used preregistered and documented structured protocols, we expected the measurement reporting for the replications to represent a high standard within the field. Any issues in measurement reporting here might suggest that other replications could face similar or greater challenges.

### Reliability

Two key aspects of measurement quality are reliability and construct validity. These can be studied using various psychometric tools [22,23]. The results of such psychometric analyses often serve as essential evidence for evaluating a measure’s validity.

Reliability serves as a pre-requisite for a valid measure in most cases, because a measure for which a participant’s response is not consistent, cannot capture a stable construct ([24]; p. 154–5). It is therefore concerning that the reporting of psychometric indicators of reliability is often insufficient [2–4,6]. Moreover, reliability reporting often limits itself to Cronbach’s alpha, a reliability indicator with strong assumptions that are themselves rarely tested [25,26]. Furthermore, research by Hussey et al. [11] has shown evidence that the reporting of reliability may not only be uncommon but also biased. They observed a disproportionate number of Cronbach’s alpha values clustered at the commonly accepted reliability threshold of .70, alongside relatively few reported values just below it. Thus, the lack of reported measurement evidence may indicate that psychometric skeletons are hiding in the closet.

As a result, a full evaluation of the reliability of the measures in our sample based only on the reported reliability is limited by the possibility of biased reliability reporting. Therefore, we also computed the measurement reliability based on the raw item data from the Many Labs original and replication studies. The data on the item responses are openly available per lab for our sample. Therefore, we can evaluate not only the reliability per measure, but also the variation in reliability across labs. The variation is relevant because Cronbach’s alpha is an indicator of reliability within a particular sample and not of the reliability of the measure in general, as it is proportional to the total variance in the target variable in the sample. Still, many researchers report and interpret Cronbach’s alpha as a universal quality of a measure [27,28]. This may lead researchers to believe that a measure that was reliable in a previous sample will also be reliable on their own, whereas it may very well not be. This can result in incorrect substantive inferences, hampering theory development. Shaw et al. [5] have already observed considerable variability in reliability in Many Labs 2, where the overall sample Cronbach’s alpha level across scales was below .5, a degree of reliability far below what many researchers would consider acceptable for most research purposes. Our second research aim is therefore to extend these findings by empirically evaluating both the degree as well as the variation of reliability of the measures in Many Labs projects 1, 2, 3, and 5.

### Construct validity and unidimensionality

The reliability of a measure cannot be meaningfully interpreted without considering how the measure’s items and the interpretation of their score are related to the construct of interest and the theory of the measurement [29]. A multi-stage process is required to statistically and substantially establish a link between the measurement and the construct of interest. For example, the way a measure’s items relate to each other and to the underlying concept (the factor relations) can differ substantially across contexts, a concept known as measurement invariance [30,31]. Thus, we cannot assume that the measure is valid in each context it is used in or meant to be used in; it is something that has to be established for each context. Besides investigating the factor structure, the measure should also be backed up substantively and logically [16]. However, what we typically observe in psychological research is that measures are rarely substantiated with validity evidence [2–4,6], and that many measures are made on the fly and reused once or not at all [32–35]. It is perhaps not surprising that most psychological measures are not fully validated, as this requires a dedicated line of research (often spanning several years), to determine the relation between measure and construct(s), and how this varies across contexts [36–38], and over time [39], among other aspects.

However, there are prerequisite checks of construct validity that researchers can conduct in most scenarios [40]. One such check is to ensure that the items measure the intended number of constructs. Often the scores on a measure or subscales of a measure are aggregated into a singular index, which is then included as a variable in a statistical model. In that case, the measure should be unidimensional. Otherwise, the researcher is interpreting a set of scores to be one concept when in fact they are multiple distinct concepts. Additionally, most reliability indicators — including Cronbach’s alpha — cannot properly estimate true variance when it is spread across distinct constructs and will result in an inaccurate estimate of reliability [25,28]. Thus, unidimensionality checks offer researchers a practical way to test whether one hurdle for construct validity has been cleared.

Shaw et al. [5] checked the dimensionality of the measurements from Many Labs 2 [19] and while some support for unidimensionality was present, they found that none of the scales in their sample met all their criteria for unidimensionality. Because the Many Labs replications reused existing measures across contexts, Shaw et al. [5] could assess validity across contexts. Our third research aim is to extend these findings. We will perform our own checks of unidimensionality on measures from Many Labs 1, 2, 3, and 5, including a check for measurement invariance across labs and conditions.

The combined goal of our three research aims is, first, to provide a descriptive account of the measurement in the included Many Labs study pairs based on the reported information. Second, we add our own assessment of reliability and unidimensionality, providing an additional source of evidence on construct validity that is not influenced by the reporting practices of our sample.

### Disclosures

We preregistered data collection, coding protocol, and planned analyses: https://osf.io/jgxyu. We deviated from our coding protocol as explained further below. The results from our preregistered analyses are described in Supplementary Analyses A. In the main text, we focus on the descriptive results. This manuscript was created in RStudio (*v2026.7.0.139*; [41]) with R Version 4.5.2 [42], and generated using the Workflow for Open Reproducible Code in Science (*v0.1.20*; [43]) to ensure reproducibility and transparency. We report how we determined all data exclusions, all manipulations, and all measures in the study. Our sample size was predetermined by the number of studies in the Many Labs projects.

## Method

### Ethics approval

This research was approved by the Tilburg University School of Social and Behavioral Sciences Ethics Review Board (under TSB_TP_REMA06).

### Data source

The data consists of three main sources: replication datasets, replication protocols, and original study articles. We retrieved the data on the preregistered replication protocols, and replication datasets from Many Labs 1, 2, 3, & 5 [17–20] from their respective OSF pages: https://osf.io/wx7ck/, https://osf.io/8cd4r/, https://osf.io/ct89g/, and https://osf.io/7a6rd/. We excluded Many Labs 4 [21], as there were no publicly available replication protocols. Additionally, we excluded the replication of Crosby et al. [44] in Many Labs 5, as it made use of videos and eye-tracking measures, which did not match this study’s focus on item-based measures. We skimmed both the replication protocols and replication datasets to ensure that they were the correct files to code measurement reporting information from. However, no coding or analysis of either of them had taken place before the analyses were preregistered. We accessed data on the 5th of May 2023, and we had no access to information that could be used to identify individual participants during or after data collection. Further details on the search strategy can be found in the coding protocol information file in the supplementary materials (URL: https://github.com/CasGoos/measurement_and_replication/tree/master/SupplementaryMaterials/CodingProtocols).

### Replication datasets

The replication datasets refer to the publicly available datasets containing the data obtained in all labs of each Many Labs replication. For the analyses, we extracted the scores on the items of each previously identified measure that also met our inclusion criteria specified in the paragraph below. If we could not clearly identify scores based on the information available in the dataset and the replication protocol, any available codebooks, analysis scripts, or study materials were used to identify the relevant scores.

The measure had to be a scale of multiple items to be included as part of our analysis of reliability and factor analyses. If cleaned data were available, we chose these over raw data, to ensure that variables were coded as intended (e.g., no reverse-coded items). We omitted pilot data from our analyses. Applying these criteria – together with cases where we could not definitively determine which dataset variables corresponded to the measure’s items – left us with a final sample of item score data from 19 replication sets, spread across approximately 35 lab locations on average.

### Replication protocols & original articles

The replication protocols refer to the publicly available protocols describing the background, methodology, and analysis of the replication of an original study. We retrieved these from the Open Science Framework (OSF) pages of the Many Labs projects (the search strategy and OSF file locations can be found in the data retrieval information supplementary document; URL: https://github.com/CasGoos/measurement_and_replication/blob/master/SupplementaryMaterials/data_retrieval_information.Rmd). We identified and retrieved all original study articles using the citations for these articles in each replication protocol.

### Unit of analysis

Our unit of analysis is a measure of a single variable within a replication protocol or original article that was used in the main analysis of the replication. For example, if conscientiousness and agreeableness were both measured using the Big 5 Personality Test, each of which was a variable in a replicated effect, then both the conscientiousness and agreeableness part of that questionnaire would be coded as their own unit of analysis. We allowed for multiple variables to be measured per study. We used the replication protocols to identify the measure of each variable. We did not include acquiescence bias checks, manipulation checks, pilot test measures, and measures added for exploratory analyses. Our final sample size was 77 measures of unique variables for both original and replication studies. Initially, the original articles contained three more measures of unique variables than the replication protocols. This difference was due to the way that the moral foundations questionnaire was framed in the original article [45] compared to in the replication protocol for Many Labs 2 [19]. In the original article, it was framed as measuring five different moral foundations, while in the replication protocol the measure assessed the two overarching categories that were used to test the main effect in both the original and replication research. The measurement information reported was comparable across all five categories, and thus we deemed that the measurement could be reduced to reflect two overarching categories to facilitate easier comparison between measurement in original and replication.

### Coding of articles and replications

#### Measurement reporting.

We evaluated the transparency of the measurement reporting practices within the original articles and replication protocols using our preregistered coding protocol containing 23 reporting practices. The criteria were based on Table 1 presented in Flake et al. [7], listing what measurement information to report. We coded a criterion as “true” if the relevant measurement information was clearly reported, “false” if it was missing or unclear, and “not applicable” if it was irrelevant for that measure (e.g., reporting factor analysis results for single-item measures). These criteria for transparent reporting practices can be seen as contra-indicators of Questionable Measurement Practices. When all criteria are coded as “true”, we consider the article’s reporting to be “full”, in that the necessary information for both evaluating and reconstructing the measure is reported. We grouped the criteria into five categories (Definition, Operationalisation, Selection/Creation, Quantification, Modification), each representing a different element in measurement reporting. Selection and creation of a measure share a category because the criteria for selecting a measure are similar to those for creating a new measure. Examples of criteria are: “The administration format (pen-and-paper/computer) and environment (in public/in a lab) are described” (Operationalisation); “The number of items are described” (Quantification). The full coding protocol can be found in the Revised Coding Protocol supplementary document (URL: https://github.com/CasGoos/measurement_and_replication/blob/master/SupplementaryMaterials/CodingProtocols/Measurement_Error_Reporting_Revised_Coding_Protocol.pdf).

**Table 1:** Proportion of measures that met each of the criteria, for both original and replication studies. Proportions are calculated based on the total number of measures to which an item was applicable (in brackets).

Categories	Criterion	Original	Replication
Definition	Measured variable is defined	0.79 (70)	0.53 (70)
Operationalisation	Exact version of the measure is specified	0.30 (77)	0.78 (77)
Operationalisation	Administration format and place are described	0.65 (77)	1.00 (77)
Operationalisation	Administration procedure is described	0.73 (77)	0.97 (77)
Operationalisation	Implemented operationalisation is justified	0.60 (77)	0.88 (77)
Operationalisation	Text or supplement has example items	0.51 (77)	0.84 (77)
Selection/Creation	Clear if the measure is newly created or not	0.48 (77)	0.04 (77)
Selection/Creation	Measure selection or creation is justified	0.48 (77)	0.90 (77)
Selection/Creation	Source of the measure is provided	0.97 (77)	0.96 (77)
Selection/Creation	Psychometric evidence from the study is given	0.23 (35)	0.15 (34)
Selection/Creation	Pre-existing psychometric evidence is given	0.24 (38)	0.08 (37)
Selection/Creation	Reliability is reported	0.34 (38)	0.11 (35)
Selection/Creation	Factor analytical results are presented	0.05 (41)	0.00 (37)
Quantification	Number of items is described	0.88 (75)	0.92 (77)
Quantification	Number of response options is described	0.86 (69)	0.90 (73)
Quantification	Recoding of responses is described	0.52 (23)	0.50 (18)
Quantification	Creation of the index is described	0.52 (48)	0.57 (44)
Modification	Administration format changes are mentioned	- (0)	1.00 (19)
Modification	Administration format changes are justified	- (0)	0.86 (7)
Modification	Translations are mentioned	1.00 (3)	0.96 (27)
Modification	Translated measures are justified	1.00 (2)	0.85 (13)
Modification	Change in N items/response options mentioned	0.83 (6)	0.86 (14)
Modification	Change in N items/response options are justified	1.00 (3)	0.58 (12)

Included within the list of measurement reporting information is, crucially, the reported reliability coefficient and type of index (Cronbach’s alpha, test-retest correlation, inter-rater reliability coefficient, etc.) when present. Also coded was the presence of any psychometric convergent, discriminant, predictive or factorial validity evidence for the measure.

After the initial coding, we made minor alterations from the preregistered coding protocol for 14 of the 23 measurement reporting practices. We revised these coded criteria because early results indicated that our original criteria were too stringent. For example, in the initial protocol, an example item of the measure had to be present within the article or protocol itself, for the measurement practice to be considered clearly reported. In the revised protocol, we also considered references to online appendices with example items sufficient. The analyses, tables, and figures presented in this article are all based on the revised coding protocol. The equivalent measurement reporting descriptives obtained with the initial protocol can be found in Supplementary Analyses B (URL: https://github.com/CasGoos/measurement_and_replication/blob/master/SupplementaryMaterials/SupplementaryAnalysesScripts/Supplementary_initial_QMP_ratio_table.rmd).

We initially intended to construct an index from the transparent measurement reporting criteria and perform regression analyses on this index and associated replication outcomes. However, we could not validate this index properly, due to the lack of reported information, including a suitable outcome to assess the index’s predictive validity. Results based on this index would be misleading to present here in the main article. A more detailed explanation of what we omitted and why, as well as the results from these preregistered analyses can be found in Supplementary Analyses A (URL: https://github.com/CasGoos/measurement_and_replication/blob/master/SupplementaryMaterials/SupplementaryAnalysesScripts/Supplementary_exploratory_version_pre-reg_analyses.Rmd).

#### Calculating reliability.

We calculated internal consistency reliabilities from the item responses on each multiple-item measure for each lab separately using available data. We calculated both Cronbach’s alpha, as well as its standard error using formulas 2 and 3 from Duhachek and Lacobucci [46]. We used these values to conduct a meta-analysis on each measure’s reliability, also referred to as a Reliability Generalization (RG) Meta-Analysis [47–49]. We performed the RG Meta-Analysis using the *rma* function from the *metafor* R package (*v5.0-1*; [50]) and default settings. We then used the results from the meta-analysis to evaluate the heterogeneity using the tau statistic and the Cochran’s Q-test [51]. We note that these indicators have low power to detect heterogeneity when within study sample sizes are small [52,53]. Therefore, we also present the 95% prediction interval (the interval within which a measure’s Cronbach’s alpha is expected to fall if calculated again within the same population) and implore that the heterogeneity results should be viewed critically [54]. We implemented no correction for publication bias because the Many Labs replications were guaranteed to be published regardless of their outcomes.

Our analyses will focus on Cronbach’s alpha, as it is the most reported reliability indicator. This enables us to compare the reported reliabilities to our calculated reliabilities. Furthermore, we calculated the standard errors of Cronbach’s alpha to study the variation in reliabilities across labs using the same measure within the RG meta-analysis. However, Cronbach’s alpha comes with strong assumptions on the underlying factor structure, including unidimensionality of the underlying factor structure. Therefore, we also estimated McDonald’s omega for each lab from the same set of replications as for Cronbach’s alpha, since it has been argued to be a more informative measure of reliability than Cronbach’s alpha with less strict assumptions [25,55]. The results based on McDonald’s omega can be found in Supplementary Analyses D (URL: https://github.com/CasGoos/measurement_and_replication/blob/master/SupplementaryMaterials/SupplementaryAnalysesScripts/Supplementary_Omega_analyses.rmd).

#### Unidimensionality.

For every measure we checked, the replicators treated it as an index of a singular latent variable. Unidimensionality therefore presents a valuable indication of a measure’s construct validity in our dataset. To assess unidimensionality, we fitted a multi-group single-factor model on the item responses from measures with suitable data — meaning at least 3 items that are approximately normally distributed — grouped by lab and any conditions or demographics that were used to test the effect in the Many Labs replications. For example, if the measure were used in ten labs across two experimental conditions, we would have twenty groups in factor modelling. We will analyze the (non-)invariance of the unidimensional factor models across the groupings of labs, and, where applicable, across the conditions and demographic variables. We evaluate if measures are consistently unidimensional across contexts, by comparing how many of our unidimensionality checks are met when structural invariance, weak (or metric) invariance, and strong (or scalar) invariance are imposed across the groupings.

Our inference of unidimensionality is based on a set of three model fit indices and their commonly used thresholds indicating adequate fit: RMSEA (threshold: < .08), SRMR (< .08), CFI (> .90). We also used the exact fit test to test if the model implied covariance matrix for the configurally invariant model matches the data variance covariance matrix tested for measurement invariance across groups against the .05 level. Additionally, we tested with a likelihood ratio test (LRT) if the fit of the weak invariant model significantly differed from the fit of the configurally invariant model, and the fit of the strongly invariant model from the weak at the .05 level. We consider the use of these thresholds sufficient for our descriptive aim, even though we understand apprehension against rules of thumb when used to evaluate measurement in individual studies. For all the included results, the power to detect measurement non-invariance in the intercept in a third of the items for half of the groups was at least .80; a prerequisite based on earlier invariance testing in Maassen et al. [1].

As a fifth additional fit index, we ran a parallel analysis for the same measures as used in the reliability analyses for each lab, to simulate the experience of a researcher in a lab determining the dimensionality of the scale in their study. A parallel analysis runs multiple exploratory factor analyses where the number of factors in the model is increased by one until the number of factors is one less than the number of items. It then compares the eigenvalues (an indication of how much variance is explained by that factor) of each factor to the eigenvalues for that factor if the data matrix was effectively random. If only the first factor has an eigenvalue that is significantly higher than the eigenvalue when the data matrix is random, the test is passed. We chose a combination of indices to test unidimensionality, since each has their own limitations, and combining them gives us a more robust picture of the unidimensionality of the measures.

These unidimensionality checks do not fully assess the validity of the measures in our sample, but rather check unidimensionality as a prerequisite for validity. Overall, the results of these checks might indicate potential limits of the validity of our sample of psychological measures. The results also function as a caveat to our assessment of reliability since Cronbach’s alpha — as an estimate of reliability — assumes unidimensionality.

## Results

### Measurement reporting

#### Transparency and Reusability.

We coded 77 measures used across the original and replication studies. Table 1 lists the prevalence of different transparent measurement reporting practices in original and replication studies. Specifically, we calculated the proportion of measures that transparently reported on the item compared to the total number of measures to which the item was applicable.

For the description of the results, we highlight the reporting practices most relevant for reusing the measure in future research, as well as the reporting practices related to the “Modification” category to understand the relation between original and replication studies.

For the description of the results, we highlight the reporting practices most relevant for reusing the measure in future research, as well as the modification reporting practices to understand the relation between original and replication studies. It was not always clear if the measure already existed or not (48% original (N = 77); 4% replication (N = 77)), which also implies that in these cases we were unsure whether the same measure was used in original and replication studies. While studies usually reported the number of items (88% original (N = 75); 92% replications (N = 77)) and response options (86% original (N = 69); 90% replications (N = 73)), it is notable that even such basic aspects were not always clearly reported. It was also often unclear how the responses should be recoded if at all (52% original (N = 23); 50% replication (N = 18)), and how an index was created from the items (52% original (N = 48); 57% replication (N = 44)). The operationalisation of the measure was usually reported clearly in the replication studies (format: 100%, procedure: 97%, justification: 88%, example items: 84%), but less so in original studies (format: 65%, procedure: 73%, justification: 60%, example items: 51%). The measures for which all criteria relevant for reuse were clearly reported on were 5.2% of all measures in original studies and 45.5% in replications.

We observed that measurement was modified in some way for 10% of measures in original studies. Modifications were more common from original to replication: 54.5%. For instances in which a modification occurred, a justification that the modification did not compromise validity was reported for all modified measures in original studies, and for 75% of modified measures in replications.

#### Reliability reporting.

**Fig 1:**
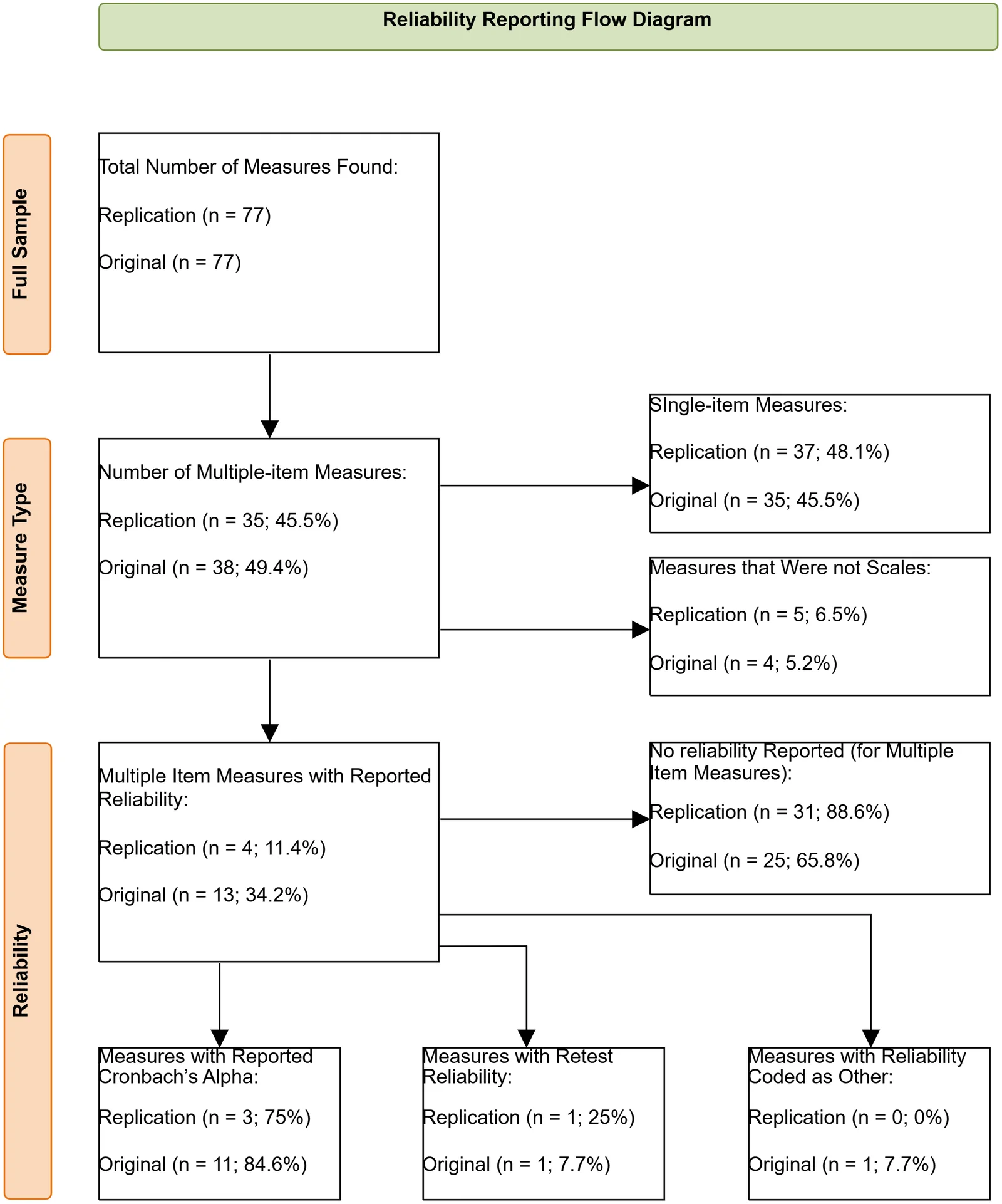
Reliability reporting flow diagram. Figure shows the number of measures as reported in both the replication protocols and original article, which meet the criterion in the box within the diagram and those criteria before it.

Fig 1 depicts a flowchart of measure types and reliability reporting in original and replication studies. First, it shows that almost half of the measures in both original (N = 35/77) and replication research (N = 37/77) were single-item measures. Second, reliability indicators were reported for multiple-item measures in only 13 out of 38 (34.2%) original studies and 4 out of 35 (11.4%) replications, which was appreciably lower than the reliability coefficient reporting percentages observed by Flake et al. [6] in their sample of original studies (60.8%) and replications (37.1%). However, in line with Flake et al. [6], we observed that reliability reporting was more common in original studies as compared to replication studies, and that Cronbach’s alpha was the most commonly reported reliability indicator in our sample as well.

#### Validity reporting.

For validity evidence the pattern was similar. Validity evidence was reported for only, eight (21.1%) multiple-item measures in original studies and five (14.3%) in replications. These proportions are slightly higher than found by Flake et al. [6] for both original (9.3%) and replication studies (6.2%). The reported psychometric indicators we could identify were two exploratory factor analyses in the original studies, and three pieces of convergent validity evidence in original studies and three in replications. Evidence for convergent, discriminant, predictive, or factorial validity from previous studies was reported in nine original studies and three replications. For single-item measures, validity evidence was reported in none of the original studies, and only two replication protocols (in both cases evidence of convergent validity).

### Analysis of item responses

#### Calculated reliability coefficients.

**Fig 2:**
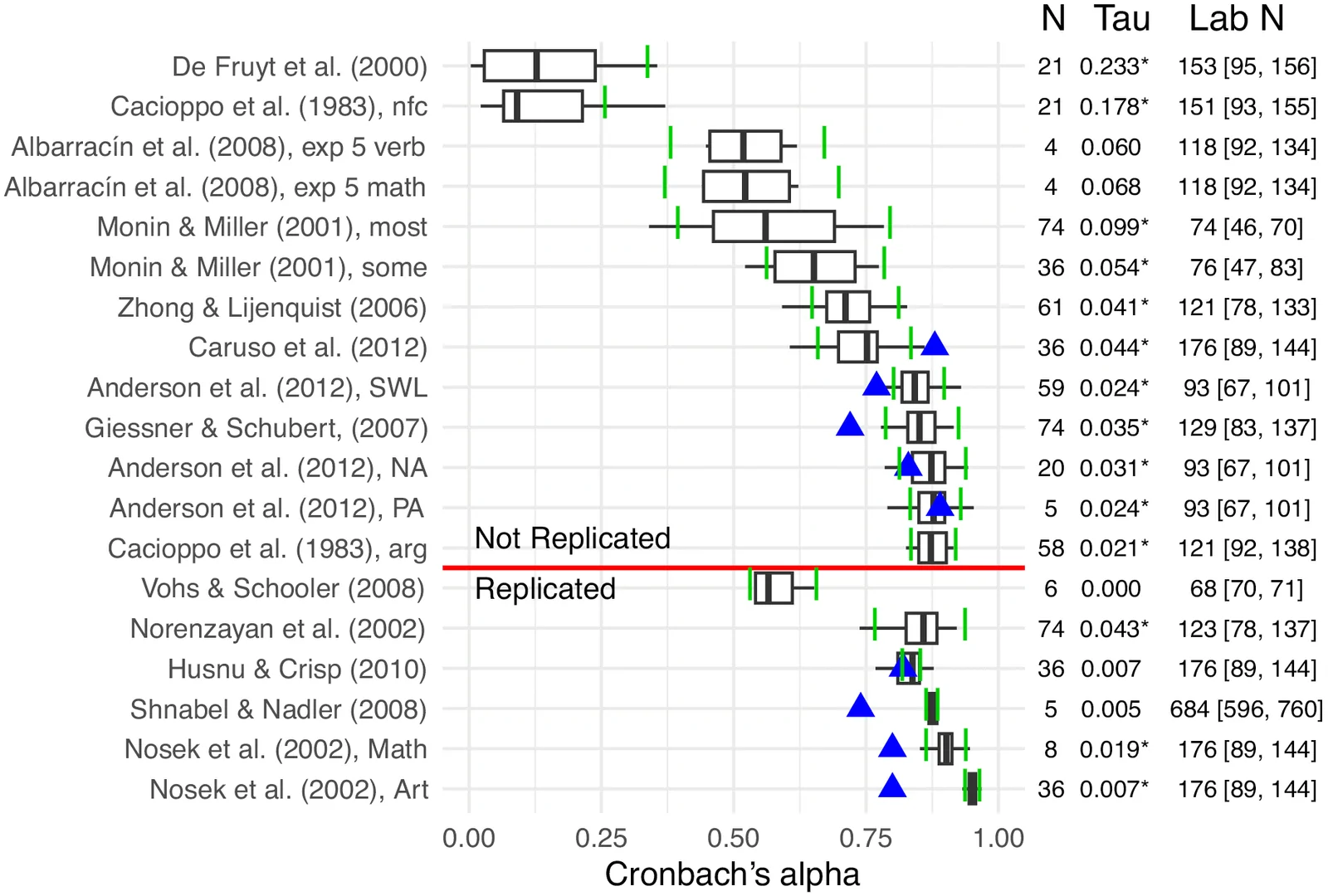
Distributions of calculated Cronbach’s alpha coefficients calculated for the responses on a measure at each lab location, across the nineteen measures for which the required raw data was available. Cronbach’s alpha values that fell below 0 were excluded. The green lines indicate the meta-analytic 95% prediction interval lower and upper bound. The blue triangles indicate the reported Cronbach’s alpha coefficient for that measure from the original article, when reported. The N column indicates the number of labs that the measure was used in. The tau column besides the figure shows the tau heterogeneity estimate based on a meta-analysis of the calculated reliabilities for each measure. Meta-analyses for which the Q-test for heterogeneity was significant at alpha = .05 are marked by an asterisk. The Lab N column shows the mean sample size per lab for that measure rounded to whole numbers, as well as the 25th and 75th quantile within brackets.

We could calculate Cronbach’s alpha for 19 measures across on average 35.3 labs, for which the required raw data were available online. The average Cronbach’s alpha coefficient across measures was 0.66 with a standard deviation of 0.32. Fig 2 displays the distributions of the calculated Cronbach’s alpha scores from each lab for each measure, horizontally separated for successful and unsuccessful replication, based on the meta-analytic p-value (nominal alpha = .05) for the global replication effect size estimate retrieved from the Many Labs reports.

We found statistically significant indication of heterogeneity across labs in Cronbach’s alpha for 14 of the 19 measures. However, as noted earlier, a more accurate indication of the heterogeneity can be observed through the prediction intervals. The prediction intervals generally show larger indications of heterogeneity for some of the measures with a lower average reliability and less heterogeneity for measures with higher reliability when compared to the tau test results. If we compare the reported reliability in the original study to the average calculated reliability in the replications, two things stand out. The reported reliabilities were typically lower than the average calculated reliabilities, and in this sample, reliabilities were reported more often in the original study if the measures had higher average calculated reliabilities (>.80) in the replications.

#### Unidimensionality tests.

**Fig 3:**
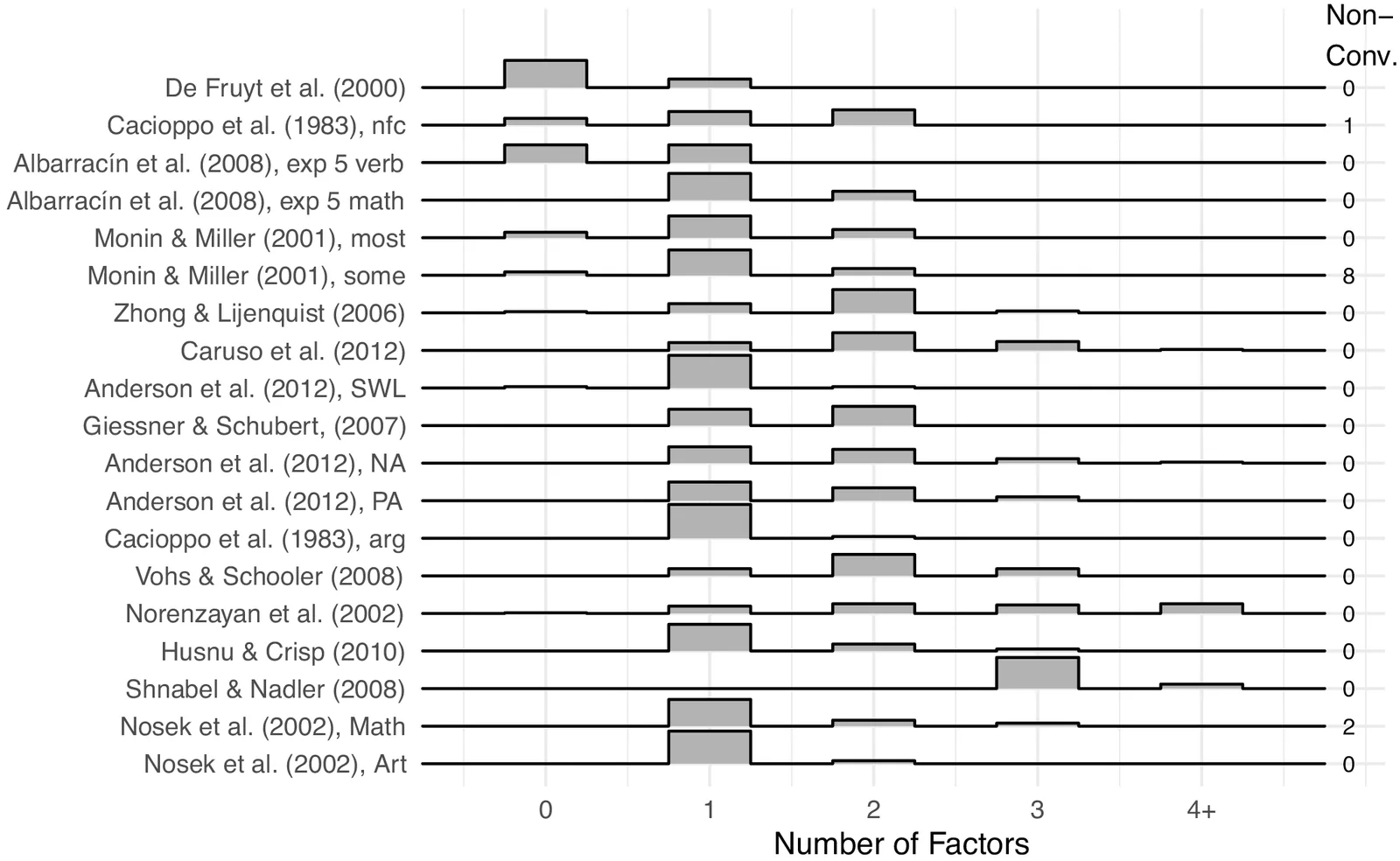
Distributions of the number of factors selected by the parallel analysis per lab. The Non-Conv. column shows the number of labs for which the parallel analysis algorithm did not converge for that measure.

Fig 3 shows the result of the parallel analysis tests of 19 measures. The parallel analysis results showed inconsistency across labs. While 62.9% of labs returned a single factor solution, 22.3% returned a two-factor, and 4.7% returned three or more factors. The remaining analyses did not converge.

Table 2 shows the fit measures obtained from the multigroup CFA of the seven measures for which the model converged and power to detect non-invariance was sufficient. For each of the seven measures, the results from the unidimensionality checks are shown for models with configural, weak, and strong invariance equality constraints across labs and conditions. When a measure passed a unidimensionality check, we marked the statistic in the cell of that test for that measure in bold.

**Table 2:** Fit statistics for the multigroup CFAs on seven applicable measures. Numbers in bold passed our unidimensionality check. For the configural invariance model, the chisq, df, and p value are those obtained from the exact fit test. For the weak and strong invariances, the chisq and df are relative to the data implied covariance matrices (so the same as for the exact fit test), the chisq and df for the LRT can be obtained by subtracting the chisq and df of the preceding model from the model’s values. A significant p-value indicates significant deviation from the data covariance matrix for the exact fit tests, or worsened fit with the added invariance restriction for the LRT tests.

measure	inv.	RMSEA	se	CFI	SRMR	chisq	df	p	AIC
Nosek 2002	Conf.	0.119	0.00	0.848	**0.076**	3,185.6	1,440	<.001	172,584
art	Weak	0.11	0.00	0.825	0.105	3,947.4	1,937	<.001	172,352
	Strong	0.14	0.00	0.641	0.143	6,568.6	2,434	<.001	173,979
Nosek 2002	Conf.	0.141	0.01	**0.974**	**0.03**	395.2	144	<.001	104,668
math	Weak	0.098	0.01	**0.969**	**0.06**	656.3	357	0.014	104,503
	Strong	0.152	0.00	0.881	0.101	1,730.5	570	<.001	105,151
Shnabel 2008	Conf.	0.162	0.00	**0.936**	**0.04**	1,795.1	324	<.001	95,996
	Weak	0.135	0.00	**0.931**	**0.065**	2,071.8	499	<.001	95,923
	Strong	0.13	0.00	**0.914**	**0.077**	2,650.8	674	<.001	96,152
Husnu 2010	Conf.	0.145	0.00	**0.966**	**0.027**	1,498.2	324	<.001	105,948
	Weak	0.119	0.00	**0.964**	**0.05**	1,724.7	499	0.005	105,824
	Strong	0.12	0.00	**0.951**	**0.063**	2,345.3	674	<.001	106,095
Zhong 2006	Conf.	0.102	0.01	**0.979**	**0.027**	227.2	100	<.001	43,314
	Weak	**0.079**	0.01	**0.978**	**0.058**	309.1	176	**0.301**	43,244
	Strong	0.08	0.01	**0.968**		446.7	252	<.001	43,229
Cacioppo 1983	Conf.	0.175	0.00	0.789	0.093	6,987.5	1,120	<.001	188,006
nfc	Weak	0.166	0.00	0.762	0.125	8,005.5	1,399	<.001	188,466
	Strong	0.177	0.00	0.676	0.145	10,676.9	1,678	<.001	190,580
De Fruyt 2000	Conf.	0.111	0.02	0.839	0.099	199.1	140	0.001	6,908
	Weak	0.115	0.02	0.761	0.141	281.7	194	0.007	6,883
	Strong	0.13	0.02	0.612	0.172	390.3	248	<.001	6,884

Taken together, our factor analytic tests for unidimensionality showed that while all measures were unidimensional in at least one of the labs based on at least one of the fit measures, we did not observe evidence of consistent unidimensionality across the labs and groups, as reflected by the overall poor fit indications under configural invariance. Within the parallel analyses, we observed that all measures but one had at least one lab that obtained a unidimensional factor solution, and every measure had at least one lab that that did not. Based on the CFA, the degree to which the model was unidimensional depended on the type of measurement invariance modelled, and the fit statistic of interest. For the configurally invariant multi-group models based on the exact fit test, all the unidimensional models rejected the hypothesis that the model implied covariance matrix matches the data variance covariance matrix which is not surprising given our total sample size and number of groups. According to the SRMR, five measures fitted well, while the CFI reached sufficient fit levels only for four of the measures. None of the measures fitted well according to the standard rule of thumb threshold for RMSEA. When testing weak invariance, RMSEAs and AIC improved in all but one measure, while this restriction led to deterioration of fit according to CFI and SRMR in all measures. The LRT test showed worsened fit due to the invariance restriction of factor loadings for all but one measure. For strong invariance, the fit worsened compared to weak invariance based on RMSEA for six measures, for all measures based on the CFI and SRMR the fit worsened, for AIC for all but one measure the fit worsened, and with the added restriction of invariant item intercepts the LRT test indicated that fit for all measures significantly deteriorated. In other words, none of the measures with any restriction of invariance met all measures of fit, and the fit was worse across all fit indices for all measures in the strong measurement invariance restricted model compared to the configural invariance model.

## Discussion

Valid and reliable measurement is crucial to study psychological phenomena robustly, but previous research has highlighted that substandard measurement and poor reporting of measures is common in psychological studies. In this study, we extracted measurement reporting information from 77 measures, reported in original and replication studies from the Many Labs projects, and calculated indices for reliability and unidimensionality based on available item response data. We found that information needed to reconstruct the measure was rarely reported in full, especially in original studies. Additionally, evidence on reliability and validity of measures was often not reported in these sets of impactful publications. Finally, recalculated Cronbach’s alpha and unidimensionality indices showed variability across measures and contexts. This further highlights the importance of transparent reporting on measurement, as the reliability and unidimensionality of a measure in one context are not a guarantee that the measurement will be sufficient in any other context. Thus, researchers should make sure to assess reliability and unidimensionality in their study as well.

As in Flake et al. [6], many studies did not fully report sufficient information to enable other researchers to reconstruct the measure. In original studies, administration format and procedure was not accurately described for 35% and 27% of applicable measures; the number of items and response options were unclear for 12% and 14%; and details on how an index was calculated, item recoding, and example items were omitted for 48%, 48%, and 49% respectively. One notable difference compared to Flake et al. [6] is that overall replications provided more complete and transparent measurement reporting than original research. We believe that this is in part due to the structured format of the Many Labs protocols, which also sometimes include specific sections for declaring deviations from the original methodology.

The measures in the replications were modified in some way in 54.5% of cases. Given how common modifications are, it is important to report them explicitly and document reliability and validity in replications. While minor differences are inevitable, substantial changes call into question whether these studies can still be considered “direct” replications. For example, the conscientiousness measure used for the replication of De Fruyt et al. [56] was reduced from the original NEO-PI-R 48-item measure to a two-item measure in the replication. It is highly unlikely that a measure with such a meaningful change would have a comparable validity and reliability to the original.

Corroborating earlier meta-research [2–6], we found that most measures used in original and replication studies lacked a reported reliability coefficient or validity evidence in the form of results from a factor analysis or convergent validity. We note that around half of the measures in our sample of studies were single item measures. While it is possible to check the reliability and validity of single item measures, these methods often require multiple measures or studies to allow for validation [57,58], an effort that is rarely taken.

For example, none of the single-item measures in our sample of original studies reported any validity evidence, and only 2 in the replication studies did, in both cases convergent validity. Even among multiple item measures, reporting issues persisted. Here we observed that reliability and validity were less commonly reported in replications than in original studies. This could be because replications implicitly defer the responsibility of demonstrating the validity of the measure to the original study. However, as we noted before, and as our reliability and unidimensionality analyses demonstrate, a measure’s reliability and factorial structure can vary across contexts. Because measurement problems could affect replication outcomes, it is important to report this information for replication studies as well. However, since the replication protocols were written before data collection, it is understandable that this information was not included there. Still, we were unable to find these details in the available supplementary materials or in the Many Labs reports.

It is concerning that most studies neither assessed nor discussed the reliability of their measures (through internal consistency) or their validity (through convergent, discriminant, or predictive validity or the factorial validity checks). Readers are left in the dark as to whether any measurement in these studies is valid or not. In the best-case scenario, the researchers assessed the reliability and validity of the measurements and found them to be sufficient, but simply did not report this often. However, it is more likely that most researchers did not check reliability and validity. In that case, we are at another crossroads: is measurement generally valid and simply unchecked, or does a genuine lack of validity remain unnoticed?

Unfortunately, given the complexity of validating a measure, it is difficult to say with certainty which scenario we are in. This is beyond the scope of this article and inconsistent with our analytical approach. Furthermore, the Many Labs projects did not specify their samples to be powered and representative for complete measurement validation. The low per lab sample size and few items — common in the replication measures — add sampling error to our reliability coefficient estimates and would bias the estimates of fit for our unidimensional factor analysis models downward. Furthermore, for the reliability assessment, even under measurement invariance and valid measurement, we would expect different reliabilities across groups simply because of differences in the true factor between groups. We cannot currently determine how much each of these contributed to our findings. Still, our item response analysis, together with existing literature, indicates that measures are often not assessed for validity and regularly fail basic psychometric requirements for valid use.

We observed that most measures passed some but not all of our unidimensionality checks and that there was considerable variability in reliability among labs, particularly for measures with low average reliability across labs. Some measures showed poor fit, while others showed good fit based on our tests – though not consistently, and fit also depended on invariance constraints imposed across groups and labs. While sufficient validity and measurement invariance are achievable, they are not for every measure in our sample. Shaw et al. [5] in their analyses of the Many Labs 2 came to similar conclusions regarding the widely ranging reliabilities and inconsistent unidimensional fit. This could mean that if any of the individual labs in the Many Labs projects checked the validity and reliability of their measure before analyzing their results, they might have concluded that their measurement failed to accurately capture the construct of interest, thereby complicating the substantive conclusions. Similar issues in measurement invariance have been noted by Maassen et al. [1].

Previous research has shown an excess of reported Cronbach’s alphas around the commonly acceptable threshold of .70 [11], demonstrating potentially biased reporting. In line with this, we observed for our measures that when an original study reported a Cronbach’s alpha, the corresponding replications typically showed relatively high reliability. Meanwhile, when the original study did not report Cronbach’s alpha, replication reliability was generally lower than when it was reported. However, while this narrative would explain why we may observe a lack of reliability and validity reporting, our observation of biased reporting is based on only a small set of measures [19] for which we could calculate Cronbach’s alpha, and is therefore too limited to support this claim with certainty. We do, however, consider it a plausible explanation for at least part of the underreporting observed here and elsewhere, and encourage future research to investigate this further using a more suitable sample.

Our intent is not to specifically criticize the Many Labs replications nor these original studies but rather to use them as illustrations of the difficulties in establishing a measure’s reliability and validity. Regardless, we would argue that full transparent measurement reporting is a responsibility of both original studies and replications. Based on our results and earlier research [1,5,11], there is reason to doubt the reliability and validity of many psychological measures, especially when they are used in a new context without transparent reporting or results from standard, readily applicable psychometric tests of reliability and factorial structure. Essential information on the reliability and validity of measures across contexts is often not reported despite the real possibility that the measures fail to meet basic psychometric standards that are relevant in understanding the robustness of psychological phenomena in different contexts [1]. Even though validating measures is challenging and resource intensive, its necessity cannot be ignored. Use of unvalidated measures might invalidate substantive interpretations drawn from both original and replication studies.

### Limitations & future research

The original studies and replications of the Many Labs projects may not be representative of typical original or replication research in psychology. Although we maintain the Many Labs projects are a relevant and high-quality source of studies, it may represent a standard that is not common throughout the field. Furthermore, the sample size considerations and setup of the Many Labs projects were not specifically targeted to measurement validation. Future research may wish to look at more representative, recent, and measurement validation oriented studies.

Another limitation inherent in our data is that replication protocols are typically shorter than research articles, which may prevent full reporting of measurement details and complicate direct comparisons of reporting practices. We believe, however, that protocols and articles remain comparable for three reasons. First, research articles are also often restricted in the space available for reporting measurement details [59,60]. Second, our revised coding protocol allowed certain items — such as reporting example items — to be fulfilled via the content in the supplementary materials. Third, beyond the protocols, few other files contained measurement details, meaning the protocol represented most of the available information, just as an article does for the original study. In other words, if a detail was missing from the protocol, it was unlikely to be found elsewhere. A difference between research articles and protocols we did not resolve was that the protocols were written before data collection. This means the protocols cannot report measurement information derived from the data, such as reliability coefficients and psychometric validity information. Future research could examine replications that do provide such data to allow more direct comparisons.

There were additional sources of information we could have included. We could have used data from original studies to recalculate reliability and validity indicators to compare with the replication results. However, it is unlikely that a substantial number of studies would have shared their data (many were conducted before the OSF and other Open Science initiatives were launched). This is likely different for more recent research [61,62].

We originally planned to formally test the difference in reported reliability between original and replication studies, and the relation between reliability and replication outcomes. However, due to the small number of reported reliabilities and measures for which reliability could be calculated, we were unable to perform these tests with sufficient power. Importantly, this limitation itself highlights a key finding: the inability to perform our preregistered analyses underscores the widespread lack of measurement information reporting in our sample.

## Recommendations

We see two key issues to address for better measurement practices: the common use of measures without established validity and the lack of transparent measurement reporting. Unfortunately, fixing these issues requires considerable time and resources. Fully validating a measure requires a lengthy and resource intensive research program spanning across years, a practice that understandably occurs only rarely beyond a handful of common (clinical and personality) questionnaires [34]. Meanwhile, improving transparency in reporting is an issue that many have tried to address for other aspects of academic articles such as preregistration deviations [63], and constraints on generality [64], but widespread success has remained elusive. Despite these challenges, we remain optimistic that even small interventions can accelerate improvement. First, these two issues are interdependent. Improving transparency in measurement reporting can in turn facilitate validation efforts. Moreover, even incremental progress, such as establishing validity for a subset of frequently used measures, can meaningfully enhance the overall quality and credibility of psychological research.

Thus, our first recommendation is that measurement reporting standards should be given greater prominence in psychological science. We found that reporting information on reliability and validity was the exception rather than the rule, and even basic information such as how many items were in the measure was reported with little consistency in our sample. The American Educational Research Association [65] guidelines exist to guide researchers in the creation and validation of scales, but not how to report the measurement details when in use. The APA guidelines [66] do address many of the same reporting practices we assessed. However, our findings and those of other research show that uptake of these standards is still minimal. One aspect where standards have changed is statistical reporting [67]. Statistical reporting standards and guidelines have received a substantial push, including recommendations from the American Statistical Association [68]. Journals have increasingly adopted statistical reporting guidelines. Although, in practice, guidelines on statistical reporting vary in their content and enforcement [67]. Still, any improvement in measurement reporting standards is valuable. Therefore, a similar high-profile push for transparent measurement reporting is at least as warranted as efforts to improve scientific robustness or statistical reporting, given that measurement data form the foundation of nearly all empirical quantitative research in psychology. We advise such guidelines to make use of the existing information in the APA guidelines [66], our measurement reporting items as documented in the Revised Coding Protocol, and to include cautionary statements against using single-item measures without relevant validity evidence. Moreover, researchers should report and evaluate measures using more informative indicators. Cronbach’s alpha alone provides limited information on the scale quality and relies on strong assumptions [26,27]. Factor-analytic evidence, such as an assessment of unidimensionality, and McDonald’s omega offer more informative alternatives and should be reported alongside or instead of Cronbach’s alpha.

Our second recommendation is that the scientific community should focus on creating, reporting, and reusing measures and measurement data in a way that allows systematic validation. To do so, measures first need to be reusable, which requires transparent measurement reporting. Researchers should then reuse existing validated measures rather than creating new, unvalidated ones. To support this, Elson et al. [33] proposed an open repository of measurement protocols to facilitate the discovery of measures and building an evidence base. They also recommended that journals should implement the Standardisation Of BEhavior Research (SOBER) guidelines to address flexibility and norming in measurement, ensuring comparability across studies. Finally, when a measure is reused across different contexts, either the raw item response data, item covariance statistics, or factor analysis output should be shared to enable other researchers to determine in which contexts, if any, the measure is valid.

Finally, we recommend that researchers seeking to replicate a study first evaluate the measurement of the original study before proceeding. Reliable and valid measurement is essential for informative replication. If the original measures are unreliable, discrepancies between the original and replication results become more likely, thereby reducing the interpretability and value of both studies. Moreover, repeating a study that relies on invalid measures does little to advance substantive knowledge and instead risks perpetuating misleading or meaningless findings. Furthermore, to make meaningful replication possible, original studies must report sufficient measurement details to allow others to reconstruct the instruments and procedures used. Without such transparency, it is impossible to know whether differences between the original and replication studies reflect true effects or merely differences in measurement. When the original measures are unreliable, invalid, or insufficiently documented, we recommend that researchers instead use their resources to conduct a replication of a study that does have reliable, valid and well-documented measurement. When replicating another study is not an option, we advise the replicating researcher to first attempt a conceptual replication using a validated measurement. Otherwise, the results of the replication cannot be confidently linked to a psychological phenomenon, undermining its contribution to theory testing [14,69]. Afterwards, a direct replication can be performed based on the conceptual replication to further assess the robustness of the effects.

## Conclusion

Cumulative knowledge on psychological phenomena starts with our ability to accurately measure the constructs of interest. For this, we need valid and reliable measurement. Yet, in our sample of Many Labs replications and original studies, reliability and construct validity evidence were rarely reported – and when reported, our analyses suggest it was often insufficient for some measures across a concerning number of contexts. Furthermore, poor transparency in measurement reporting hinders the reuse of existing measures. Our findings and those of a growing body of literature highlight that changes in both the use and reporting of measurement are necessary; without them, psychological effects lack the measurement foundation needed to justify their connection to true phenomena. Fortunately, even small improvements — in the adoption of measurement reporting guidelines, in data and materials sharing, and in treating valid measurement as a prerequisite to substantive interpretation and inclusion in replication projects, especially for single item measures — can spark the proliferation of validated measurement.

## Supporting information

Supplementary analyses A (HTML)

Supplementary analyses B (HTML)

Supplementary analyses C (HTML)

Supplementary analyses D (HTML)
