# Supplementary material for: Measurement reliability, construct validity, and transparent reporting in original and replication psychological research
Source: PLoS One. 2026 Sep 2;21(9):e0356070. doi: 10.1371/journal.pone.0356070 (PMC13537538; doi:10.1371/journal.pone.0356070)
Supplement: Supplementary file 1 [file pone.0356070.s005.docx]

Response to Reviewers

Dear Editor,

We would like to thank both you and the reviewers for the insightful comments. We have revised our manuscript ,to improve itsclarity and the nuance in our interpretation of its results.

Specifically,We also changed our unidimensionality tests to be more appropriately suited to the data and revised the interpretation of our results to be more cautious and placed them more clearly within the larger context of the literature.

We address all comments point by point below, where our replies to the reviewers are in **bold**, and copied sections from the revised manuscripts are in ***bold italics*** and in quotation marks.

We thank you for considering our manuscript for publication in PLoS One and are looking forward to reading your thoughts.

Your sincerely, also on behalf of my co-authors,

Cas Goos

## Reviewer #1:

This is an interesting and well-conducted study addressing an important issue in psychological research, namely the transparency and quality of measurement reporting. The focus on both original and replication studies, particularly within the Many Labs framework, provides a strong and relevant basis for evaluating current practices in the field. The use of openly available data to assess reliability and unidimensionality further strengthens the methodological rigor and contributes to the credibility of the study.

The findings are presented nicely and provides valuable insight into persistent shortcomings in reporting reliability and validity. The discussion appropriately situates the results within the broader literature on the replication crisis and research transparency, and the practical recommendations provided are both relevant and useful for improving future research practices.

**REPLY: We would like to thank the reviewer for their positive evaluation of our manuscript, and have sought to ensure that these elements have retained their central place in our manuscript.**

I am a little concerned about a few areas to improve the readership value of the manuscript. For instance, providing more detail on the criteria used to define “full” measurement reporting would improve transparency.

**REPLY: We agree that defining this more explicitly would improve clarity. We have changed the relevant sections as follows:**

**Page 12 added: *“When all criteria are coded as “true”, we consider the article’s reporting to be “full”, in that the necessary information for both evaluating and reconstructing the measure is reported.”***

**Page 25 added: *“[...] many studies did not fully report sufficient information to enable other researchers to reconstruct the measure”***

**This way, the term “full” is directly related to how we measured and operationalized the reporting of measurement information.**

Additionally, a brief elaboration on the implications of unstable reliability across labs for theory development could further strengthen the discussion.

**REPLY: We have changed the relevant sections as follows:**

**Added the following sentence to Page 5: *“As a result, critically testing theories on substantial phenomena is stilted because we cannot establish that the relevant constructs were assessed (14–16).”***

**Added the following sentence to Page 7: *“This may lead researchers to believe that a measure that was reliable in a previous sample will also be reliable on their own, whereas it may very well not be. This can result in incorrect substantive inferences, hampering theory development.”***

**Added the following sentence to Page 32: *“Otherwise, the results of the replication cannot be confidently linked to a psychological phenomenon, undermining its contribution to theory testing (14,69).”***

Synchronization among the objectives, results, and discussions would be beneficial.

**REPLY: To better synchronize these elements, we have aimed to increase the cohesion by adding the following:**

**Added On Page 8 the following explicit description of the goals: *“The combined goal of our three research aims is to first provide a descriptive account of the measurement in the included Many Labs study pairs based on the reported information. We add to this our own assessment of reliability and unidimensionality to provide an additional source of evidence on the validity that is not influenced by the reporting practices of our sample.”***

**On Pages 24-25, we added the following to more clearly link the Discussion to this new formulation of the goals: *“Finally, recalculated Cronbach’s Alpha and unidimensionality indices showed variability across measures and contexts. This further highlights the importance of transparent reporting on measurement, as the reliability and unidimensionality of a measure in other contexts is not a guarantee that the measurement in any context. Thus, researchers should make sure to assess reliability and unidimensionality in their study as well.”***

**Finally, the reformulation of the unidimensionality analyses and their interpretation (detailed below) as discussed on Pages 27 is also relevant for aligning our goals, results, and discussion more closely.**

Practical implications may be given with clarity within the concluding remarks.

**REPLY: We thank the reviewer for bringing to our attention that the practical implications needed to be highlighted in the conclusion. We therefore changed the final sentence of the conclusion (Page 33) as follows: *“Fortunately, even small improvements in more widespread adoption of measurement reporting guidelines, data and materials sharing, valid measurement as a prerequisite to substantive interpretation and inclusion in replication projects, especially for single item measures, can spark the proliferation of validated measurement.”***

Overall, this is a meaningful contribution to the literature and will be of interest to researchers concerned with methodological rigor and reproducibility in psychological science. I thank the authors for addressing such a relevant agenda of research.

## Reviewer #2:

Thank you for the opportunity to read and review this paper. I read the manuscript with interest. The paper addresses an important issue in psychological science, namely the transparency of measurement reporting and the stability of reliability and unidimensionality across Many Labs studies. I believe the manuscript has potential, because the topic is relevant and the combination of reporting review and secondary psychometric analyses is valuable. At the same time, I think the paper needs substantial revision before it can be considered for publication.

My first concern is about the reliability analyses. The number of reported Cronbach’s alphas in the original papers appears quite limited, and this makes it difficult to draw strong conclusions about differences between reported, unreported, and estimated reliabilities. This is particularly relevant when, later in the Discussion, you suggest possible bias in measurement reporting. That interpretation is plausible, but the available empirical basis seems rather limited, and I am not sure the current data are sufficient to support such a conclusion strongly. I did not see formal tests, such as moderation-type analyses, that would directly support that claim, although I understand that such tests may themselves be underpowered given the small number of measures. You do acknowledge related limitations, but the point still seems somewhat too strongly promoted in the conclusions.

**REPLY: We agree with the reviewer that upon reinspection our conclusions were still too strongly worded in our discussion section, we therefore rewrote the discussion section on biased reporting to be more tentative and relate the conclusions more to existing literature, and request further research:**

**Revised** **on Pages 27-28 : *“Previous research has shown an excess of reported Alphas around the commonly acceptable threshold of .70 (11), demonstrating potentially biased reporting in Cronbach’s Alpha coefficients. In line with this, we did observe for our measures that if an original study reported a Cronbach’s Alpha, the corresponding replications typically showed relatively high reliability. Meanwhile, if no Cronbach’s Alpha was reported in the original study, the reliability in the replications was generally lower than if a Cronbach’s Alpha was reported. However, while this narrative would explain why we may observe a lack of reliability and validity reporting, our observation of biased reporting is based on only a small set of measures (19) for which we could calculate the Cronbach’s Alphas, and is therefore too insubstantial to state this claim with certainty. We do, however, consider it to be a candidate to explain at least part of the underreporting observed by us and others, and encourage future research to extend the investigation to a more suitable sample.”***

My second concern is about the unidimensionality analyses. In my view, these analyses are problematic because many labs may have relatively small sample sizes, and fitting CFA models separately within small samples can produce unstable parameter estimates and unreliable fit indices. In such cases, poor fit may reflect sample size limitations rather than genuine measurement problems. You partly acknowledge convergence problems and the possible role of small sample sizes, but I think this concern should receive more weight in the interpretation. Alternative approaches could include multigroup CFA or multilevel CFA models that explicitly account for clustering by lab, rather than fitting separate models at the lab level.

**REPLY: We thank the reviewer for their suggestion to use multigroup CFA as a more fitting method for our sample and analysis goal. It had not yet occurred to us to use this type of model, and we agree it is more suitable for our data.**

**A brief description of the model from our manuscript added on Page 14 is as follows: *“To assess unidimensionality, we fit a multi-group single-factor model on the item responses from measures with suitable data – meaning at least 3 items that are approximately normally distributed – grouped by lab and any conditions or demographics that were used in the test of the effect in the Many Labs replications. For example, if the measure were used in ten labs and there were two experimental conditions, we would have twenty groups in our comparison.”***

More generally, given the low number of studies and variables ultimately available for these analyses, I am not fully convinced that the results are sufficiently informative to support the broader conclusions currently drawn.

**REPLY: We again agree that more tentative conclusions are warranted. We have therefore also elaborated on the conclusions based on these analyses to be more clearly tentative.**

**The most extensive revisions related to this point are on Page 27 (although the tone has been adjusted throughout the article): “*We cannot currently state with certainty the degree to which any of these have resulted in the findings we observe. Still, our item response analysis in combination with existing literature indicate that measures are often not assessed on their validity and regularly fail basic psychometric requirements for valid use.***

***We observed that most measures passed some but not all our unidimensionality checks and that there was considerable variability in reliability among labs for measures with low average reliability across labs too. Some measures showed poor fit, while other measures showed good fit, albeit not consistently and abiding by invariance constraints across different groups and labs . While sufficient validity and measurement invariance is reachable, it is not a universally guaranteed quality of the measures in our sample. Shaw et al. (5) in their analyses of the Many Labs 2 came to similar conclusions regarding the widely ranging reliabilities and inconsistent unidimensional fit. This could mean that if any of the individual labs in the Many Labs projects checked the validity and reliability of their measure before analyzing their results, they might have concluded that their measurement failed to accurately capture the construct of interest, thereby complicating the substantive conclusions. Similar issues in measurement invariance have been noted by Maassen et al. (1).”***

Relatedly, I think it would strengthen the paper if you discussed more explicitly whether the current analytic strategy can recover trustworthy results under realistic conditions. A simulation study reflecting a similar scenario, with small per-lab sample sizes, group effects, and questionnaire responses, could be a useful way to test whether, under a favorable validity scenario, the current approach would still produce apparently poor reliability and fit. With small per-lab samples, observed alpha coefficients can vary substantially simply because of sampling error, and fit indices may also perform poorly. There is considerable methodological literature on these issues, so even a more explicit discussion of that literature would already help if a simulation is beyond the scope of the paper.

**REPLY: Because it is outside of the scope of the study, we have not done performed the simulation study as was suggested. Instead, we address the reviewer’s point in the following ways:**

**We added the following caveat to make the power issues explicit in the Discussion section on Pages 26-27 :** ***“Unfortunately, given the complexity of validating a measure, it is difficult to say with certainty which scenario we are in. This is beyond the scope of this article and inconsistent with our analytical approach. Furthermore, the Many Labs projects did not specify their samples to be powered and representative for complete measurement validation. The low per lab sample size and few items — common in the replication measures — add sampling error to our reliability coefficient estimates and would bias the estimates of fit for our unidimensional factor analysis models downward. Furthermore, for the reliability assessment, even under MI and valid measurement, just because of differences in the true factor between groups we would expect different reliabilities across groups. We cannot currently state with certainty the degree to which any of these have resulted in the findings we observe. Still, our item response analysis in combination with existing literature indicate that measures are often not assessed on their validity and regularly fail basic psychometric requirements for valid use.”***

**We conducted a power analysis similar to Maassen et al. (2015) on our ability to detect measurement invariance in our data, as reported on Page 14 of the manuscript: *“For all the included results the power to detect Measurement non-Invariance in the intercept in a third of the items for half of the groups was at least .80, this prerequisite was based on (1).”***

Overall, I am more convinced by the descriptive part of the manuscript than by the testing part. The descriptive results on reporting standards in original and replication studies are, in my opinion, informative and worth reporting. By contrast, the analyses of lab-level reliability and factorial validity seem more difficult to interpret, and I am therefore not sure that some of the stronger conclusions fully follow from the available evidence. For this reason, I would encourage you either to frame these analyses more cautiously or to strengthen their methodological justification.

**REPLY: We thank the reviewer for their constructive criticism of our article, and we hope that our changes mentioned above will have addressed their concerns sufficiently.**

As minor comments, I found Figure 2 difficult to read. The green lines are almost invisible, and the figure does not report the actual sample sizes or even sample size ranges for the lab-level analyses. Adding clearer visual elements and explicit information on the sample sizes would make the figure easier to interpret.

**We increased the size and changed the color of the green lines. We also added the Mean sample size per lab as well as the 25^th^ and 75^th^ quantiles of the sample size.**

The manuscript would also benefit from some language editing. A few specific examples are: “discrimant” should be corrected to “discriminant” in the Abstract (p. 2, line 27); on p. 3, lines 47–50, the sentence beginning “Psychometrics offers a wide range of tools...” is too long and difficult to follow, and “asses” should be corrected to “assess”. The sentence also finishes without the dot; on p. 8, line 168, “practical wat” should be “practical way”; on p. 17, lines 383–385, the sentence beginning “which was appreciably lower than found in Flake et al. (6). on reliability coefficient reporting...” should be revised for grammar and capitalization; and on p. 14, lines 312–315, the sentence beginning “Our analyses will focus on Cronbach’s Alpha...” contains phrasing that is currently unclear, especially “the standard errors of Alpha caused in the RG meta-analysis.” .

In sum, I think this is an interesting and potentially useful study. However, I believe the descriptive contribution is stronger than the psychometric testing component in its current form. I would therefore encourage a more cautious interpretation of the reliability and unidimensionality analyses, and a clearer alignment between the evidence presented and the conclusions drawn.
