# Supplementary material for: Measurement reliability, construct validity, and transparent reporting in original and replication psychological research
Source: PLoS One. 2026 Sep 2;21(9):e0356070. doi: 10.1371/journal.pone.0356070 (PMC13537538; doi:10.1371/journal.pone.0356070)
Supplement: Supplementary file 2 [file pone.0356070.s001.html]

Supplementary\_exploratory\_version\_pre-reg.knit


```
### NOTE: also run the setup code in the manuscript
library(lmerTest)
```

```
## Loading required package: lme4
```

```
## 
## Attaching package: 'lmerTest'
```

```
## The following object is masked from 'package:lme4':
## 
##     lmer
```

```
## The following object is masked from 'package:stats':
## 
##     step
```

```
library(betareg)
library(statmod)
```

```
# Seed for random number generation
set.seed(17042023)
knitr::opts_chunk$set(cache.extra = knitr::rand_seed)
```

```
# combine replication and original data 
is_replication <- c(rep(FALSE, 77), rep(TRUE, 77))

combi_org_rep <- rbind(coded_data_original, coded_data_replications)

combi_org_rep <- cbind(combi_org_rep, is_replication)

combi_org_rep$has_reliability <- combi_org_rep$reliability_type != "Not Reported" & combi_org_rep$reliability_type != ""


# function to calculate QMP ratios
calculating <- function(Data){
  QMP_data <- data.frame(def_ratio = rep(NA, 154),
                         op_ratio = rep(NA, 154),
                         sel_ratio = rep(NA, 154),
                         quant_ratio = rep(NA, 154),
                         mod_ratio = rep(NA, 154),
                         QMP_ratio = rep(NA, 154),
                         op_REV_ratio = rep(NA, 154),
                         sel_REV_ratio = rep(NA, 154),
                         quant_REV_ratio = rep(NA, 154),
                         mod_REV_ratio = rep(NA, 154),
                         QMP_REV_ratio = rep(NA, 154))
  
  def_count <- as.numeric(Data$def_1)
  def_count[is.na(def_count)] <- 0
  def_total <- as.numeric(!is.na(Data$def_1))
  QMP_data$def_ratio <- 1 - round(def_count / def_total, 4) 
  QMP_data$def_ratio[is.na(Data$def_ratio)] <- 0
  
  op_count <- rowSums(Data[16:20], na.rm = TRUE)
  op_total <- rowSums(!is.na(Data[16:20]))
  QMP_data$op_ratio <- 1 - round(op_count / op_total, 4)
  
  sel_count <- rowSums(Data[23:26], na.rm = TRUE)
  sel_total <- rowSums(!is.na(Data[23:26]))
  QMP_data$sel_ratio <- 1 - round(sel_count / sel_total, 4) 
  
  quant_count <- rowSums(Data[29:32], na.rm = TRUE)
  quant_total <- rowSums(!is.na(Data[29:32]))
  QMP_data$quant_ratio <- 1 - round(quant_count / quant_total, 4)  
  
  mod_count <- rowSums(Data[34:39], na.rm = TRUE)
  mod_total <- rowSums(!is.na(Data[34:39]))
  QMP_data$mod_ratio <- 1 - round(mod_count / mod_total, 4) 
  
  MP_total <- def_total + op_total + sel_total + quant_total + mod_total
  MP_count <- def_count + op_count + sel_count + quant_count + mod_count
  QMP_data$QMP_ratio <- 1 - round(MP_count / MP_total, 4) 
  
  op_REV_count <- rowSums(Data[c(18,19,41,42,43)], na.rm = TRUE)
  op_REV_total <- rowSums(!is.na(Data[c(18,19,41,42,43)]))
  QMP_data$op_REV_ratio <- 1 - round(op_REV_count / op_REV_total, 4) 
  
  sel_REV_count <- rowSums(Data[c(24,26,44,45)], na.rm = TRUE)
  sel_REV_total <- rowSums(!is.na(Data[c(24,26,44,45)]))
  QMP_data$sel_REV_ratio <- 1 - round(sel_REV_count / sel_REV_total, 4)  
  
  quant_REV_count <- rowSums(Data[c(32,48,49,50)], na.rm = TRUE)
  quant_REV_total <- rowSums(!is.na(Data[c(32,48,49,50)]))
  QMP_data$quant_REV_ratio <- 1 - round(quant_REV_count / quant_REV_total, 4) 
  
  mod_REV_count <- rowSums(Data[52:57], na.rm = TRUE)
  mod_REV_total <- rowSums(!is.na(Data[52:57]))
  QMP_data$mod_REV_ratio <- 1 - round(mod_REV_count / mod_REV_total, 4) 
  
  MP_REV_count <- def_count + op_REV_count + sel_REV_count + quant_REV_count + mod_REV_count
  MP_REV_total <- def_total + op_REV_total + sel_REV_total + quant_REV_total + mod_REV_total
  QMP_data$QMP_REV_ratio <- 1 - round(MP_REV_count / MP_REV_total, 4) 
  
  return(QMP_data)
}

# calculating the QMP ratios for each QMP item.
QMP_Ratio_data <- calculating(combi_org_rep)

QMP_Ratio_data <- cbind(QMP_Ratio_data, is_replication)
```

# Supplementary Analyses A: Pre-registration & Exploratory Analyses

This appendix contains two things. First of all the pre-registered
exploratory analyses. Additionally, it also includes the revised
analyses shared in the pre-print for this article as found in: https://osf.io/2gau8\_v1.

Every hypothesis test used an alpha significance cutoff of .05. No
correction for multiple testing was applied, to avoid inflating type II
error rate. The generally small sample size and consequently low power
of these analyses meant that a smaller alpha would increase the number
of false negatives. On top of that, these analyses were considered as
exploratory. In this exploratory context, the rate of discovery of any
potential true effects was given more value relative to decreasing any
false positives compared to typical confirmatory research. That as a
consequence obtaining false positives was more likely has been accepted,
as false negatives were deemed more harmful to this study’s aims.

Some of these analyses were also conducted after removing the
potentially confounding factor of measures for which the number of items
were reduced in the replications. These results can be found in Supplementary
Analyses C.

# Initial Analysis Goals & Process and Reasoning of Omission

We initially had planned to explore relations between both
measurement reporting and reliability with replication outcome. This
involved regression models wherein an outcome - either the replication
outcome from the Many Labs replications or our QMP indicator - and a
predictor - reliability, our QMP indicator, or whether it is an original
study or a replication. The idea of doing this was based on earlier
research that has shown that reliability is related to replication
success. The reason for this is because lower reliability introduces
more noise into our data, which leads to less stable estimates across
multiple studies of the same effects, which leads to less replicable
effects. We also wanted to check for measurement-based predictors for
replicability more broadly, which includes QMPs from the measurement
reporting side of things. The reason for doing this was because knowing
what predicts replicability can help us understand the various forces
that can inhibit replicability and inform us in when and how to choose
what to replicate. This last point is something we still explore in the
article itself, albeit using descriptive information and more
logic-based inferences rather than statistical testing.

The reason we omitted these analyses is largely for two reasons: (1)
if we want to model a relationship between two variables, we also need
to account for all potentially relevant confounders to get a good
estimate; (2) predicting replicability as a goal may not really be all
that interesting theoretically or practically. To elaborate on point 1,
what we lack in our case is a theoretical model to understand all the
various influences on replicability. Without knowing these, we could
technically still use reliability as a predictor, but our goal and
motivation to check reliability was to test the theoretical relation
between reliability and replicability in practice. The “in practice”
part becomes difficult however because practice is messy and we have to
account for that messiness, but we can’t, because we don’t know what to
account for. Trying to create a model for this or do simulations to
figure this out drifts from the main goal of the article: to understand
the reporting, reliability, and validity of measurement in original; and
addressing confounds to replication success is just simply beyond the
article’s scope. To elaborate on point 2, while there are some reasons
to predict replicability for practical reasons - mostly for deciding
which study to replicate. But as we stated in point 1, it is difficult
to effectively predict replicability in our study. So then we are left
with the potential theoretical interest of predicting replicability. The
idea here is that if we want to understand why something may not
replicate, then trying to find predictors for replicability should give
us an understanding of what makes something replicable or not. But
understanding what makes something replicable is missing the goal of why
we care for replicability, which to some extent is that we don’t care
about replicability. What we care about is getting accurate estimates of
an effect of interest, or less statistically is we want to have some
certainty that our understanding of how much a psychological phenomenon
exists - if it exists at all. But if we just look at trying to see how
we can get the most replicable effects possible that’s easy, we just
conduct Stroop-tasks and other basic psychology 101 tests and we can
replicate around the clock. Making studies replicable should not be a
goal in and of itself, what we desire is to get accurate estimates of a
phenomenon of interest.

There is another key element from the first pre-print version of the
article and the pre-registration: we developed a QMP index to allow us
to test the relation between measurement reporting and replication
outcome statistically. The QMP index was essentially the same as the QMP
ratio presented in the article, the number of QMP items that were rated
as insufficiently reported on divided by the total number of applicable
items. There were two differences, one is that we also created QMP
ratios per specific QMP category (definition, selection/creation,
operationalization, quantification, and modification); second is that we
created this QMP ratio as a variable, thus treating our QMP items
collectively as an index. Having an index means that it should be
validated in some way (a point we make throughout the article too). Now,
we did not make a measure of a construct, so construct validity was not
a concern of ours. However, an indicator should have some predictive
validity, which requires us to validate that that is the case before we
actually use the measure. We did not have the means nor the method nor
any idea on how to do this within our study in a way that would be
appropriate. We can propose that QMPs should predict replciabiltiy and
then show that this is the case, but if our analysis itself is to
investigate whether this is the case, then we can’t treat it as
validating our measure, because before doing that analysis there was no
guarantee that it should be related, and thus we can’t use observing
that relationship as evidence that the measure worked as intended.

So, what would we advice future researchers interested in probing
relations with replication and/or creating a QMP index. First, we would
advise you to only probe relations with replication if you have a
practical reason for wanting to predict replicability. If you want to
look into this relation for theoretical reasons, we would advise you to
switch your goals. Instead, try and focus on understanding what makes
for accurate and robust estimates. This is closer to what we are
actually interested in, so if there is something we should want to
understand on a deeper level, it is this. Doing so would require taking
a more meta-analytic approach to your outcomes, as that can give you
more undeerstanding of an effect’s robustness compared to the overall
p-value that we used as an indicator of replication success. Second, if
you want to create a predictve index or even just a general useful
descriptive rating scale based on reporting practices, such as QMPs, we
would advise you to first of all make this its own study (or studies) as
this is likely an involved process. Then, you may want to look at
measurement quality standards, such as the @americaneducationalresearchassociationStandardsEducationalPsychological2014
guidelines, to get something more substantially theoretical as a
background for what you mean with what should be reported about a
measure. Finally, make use of existing research on measurement reporting
such as this one (you can look at our coding protocol supplements and
the revisions we did to understand what problems may be smart to avoid),
but also @flakeConstructValidationSocial2017, @maireadshawMeasurementPracticesLargescale2020,
and @flakeConstructValidityValidity2022
have done work on this and have openly available materials. While we
made use of these materials, in our goal to make a QMP indicator, we
regret mostly (1) not deciding on a strong definition of what good
measurement reporting should in the end do, as well as (2) an outcome
that reflects the impact of good measurement reporting as we defined
it.. We therefore decided to leave creating an index behind for this
project, but hopefully this information ahs been useful to inform future
research.

Below are the results for our pre-registered analyses for those
interested. Take all of them with a big grain of salt.

## Hypothesis 1

As a first step it is worth investigating what the state of
measurement error is in original and replication research in psychology.
Thus, this study investigated the following:

- RQ1a. What is the degree of score reliability in replications of
  psychological research?
- RQ1b. What is the degree of score reliability in original
  psychological research?

Since direct replications intend to investigate effects in nearly
identical scenarios with nearly identical measures as the original
study, it is expected that measurement errors are as present in
replication research as in original psychological research.

- H1. No difference is present in the degree of score reliability
  between replication research and original psychological research.

### Analysis

There were too few reported reliability coefficients, therefore the
test for hypothesis 1 was changed from the pre-registration, and we
won’t try to do that pre-reg analysis because they are simply to few.
Thus for testing hypothesis 1, instead of comparing the reported
Cronbach’s alpha coefficient between original and replication, the total
number of reported reliability coefficients were compared instead. This
total also included reliability coefficients other than Cronbach’s
alpha. This comparison was implemented via a chi-square test using the
base R stats package [@R-base].

Reliabilities were found to be more commonly reported in original
articles compared to replication protocols (\(\chi^{2}\)(1) = 4.09, *p* = .043).
These results are contradictory to hypothesis 1.

## Hypothesis 2

While reliability estimates are generally considered a measure
specific feature, this is false. They are context dependent, and as a
result the estimates of an effect can differ across contexts [@cho2015cronbach; @pauly2018resampling]. With
large enough differences across contexts, the effect estimates between
different replication attempts could deviate from each other beyond
direct comparison. To investigate this potential problem, the variation
in score reliability is assessed for each measure when used in different
contexts.

- RQ2. To what degree do reliability estimates differ within a
  replication set of the same original study?

Even though reliability estimates are context dependent, sufficient
protocol structure can counteract the variance in reliability estimates.
It is possible to reduce variability as the result of context using
structured protocols, as was done in the Many Labs projects this study
analyzed.

- H2. There is no significant variation in the reliability estimates
  of replications of the same original study.

### Analysis

Using the lmer function from the lme4 package [@R-lme4], a multilevel null model with the
Cronbach’s alpha or omega coefficient of a replication as dependent was
specified, where the random intercept was grouped by replication set.
The formulas for this model, where \(alpha\_{ij}\) represents the calculated
Cronbach’s alpha coefficient of a single study at one lab location with
\(i = 1,..., n\_{locations}\) and \(j = 1,...,n\_{replications}\) look as
follows:

\[\begin{align}
& Level 1: alpha\_{ij} = \beta\_{0j} + e\_{ij} \qquad\qquad\quad e\_{ij}
\sim N(0, \sigma\_{e}^{2}) (\#eq:level1H2) \\
& Level 2: \beta\_{0j} = \gamma\_{00} + u\_{0j} \qquad\qquad\qquad
u\_{0j} \sim N(0, \sigma\_{u0}^{2}) (\#eq:level2H2) \\
& Mixed: alpha\_{ij} = \gamma\_{00} + u\_{0j} + e\_{ij} (\#eq:mixedH2)
\end{align}\]

The purpose of this analysis was to investigate variability in
calculated reliability coefficients within and between replication
sets.

The between-group and within-group variance for Cronbach’s alpha were
approximately 0.103 and 0.012 respectively. The resulting ICC, obtained
using formula @ref(eq:ICC), was approximately 0.894. This suggests that
variance was larger between replication sets than within. The same
analysis for omega coefficients even showed a relatively larger degree
of between group variance (between-group variance 0.002, within-group
variance 0.010, ICC = 0.844). Both results are in line with hypothesis
2, although caution in interpreting this result is advised due to the
small sample size and the lack of an available inferential test.

## Hypothesis 3

This study investigated the relationship between reliability and
replicability, by using data from real life replication projects.

- RQ3. What is the association between replication study score
  reliability and replication outcome?

Greater score-reliability means greater consistency in estimation as
variance around the estimate of the true effect is decreased.
Consequently measures across different occasions, including between
original and replication, should also become more consistent [@nunnaly1994assessment]. When original and
replication research share consistent result, and as long as the studied
effect is true, then on average their statistical conclusions should
converge.

- H3. Score reliability in replication research is positively
  associated with successful replication of an original finding.

### Analysis

In order to test hypothesis 3, the average calculated Cronbach’s
alpha level within a replication set is used as a predictor of
replication success. Replication success was either set to 1 if it
reached meta analytic significance at an alpha of .05 in the same
direction as that found in the original study, or 0 otherwise. This test
aligns with a logistic regression model. With \(i = 1,..., n\_{replications}\), \(p\_{replicated}\) being binomially
distributed, and \(alpha\) referring to
the averaged calculated Cronbach’s alpha coefficient, the following
regression equation was used to model the relation:

\[\begin{equation}
f(replicated\_{i}) = \beta\_{0} + \beta\_{1} alpha\_{i} (\#eq:H3Main)
\end{equation}\]

Where:

\[\begin{equation}
f(.) = log\frac{p\_{replicated}}{1 - p\_{replicated}} (\#eq:LogitLink)
\end{equation}\]

In this data, replication attempts at different lab locations can be
seen as nested within a replication set. In order to see if this nesting
might impact the analyses of hypothesis 3, multilevel logistic
regression models were included as sensitivity checks. The dependent
variable remains replication success at the replication set level, while
Cronbach’s Alpha is no longer averaged across locations. The models used
were a multilevel logistic random intercept and random slope model.

The results of the logistic and multilevel logistic models are shown
in Table @ref(tab:ReplicabilityReliabilityTestTable) for both Cronbach’s
alpha and omega reliability coefficients.

```
# creating the dtaframe for the table
H3_table_dataframe <- rbind(H3_test_result_main_alpha$table, H3_test_result_int_sensitivity_alpha$table, H3_test_result_main_omega$table, H3_test_result_int_sensitivity_omega$table)

# adding rows to give indication of specific sections (apa_table being awkward)
H3_table_for_printing <- rbind(c("Alpha", "", "", "", ""), c("Logistic Regression Model", "", "", "", ""), H3_table_dataframe[1:2,], c("Random-Intercept Multilevel Logistic Model", "", "", "", ""), H3_table_dataframe[(3:4),], c("Omega", "", "", "", ""), c("Logistic Regression Model", "", "", "", ""), H3_table_dataframe[(5:6),], c("Random-Intercept Multilevel Logistic Model", "", "", "", ""), H3_table_dataframe[(7:8),])


# print the table in apa formatting

H3_table_for_printing
```

```
## A data.frame with 5 labelled columns:
## 
##                                          term estimate        conf.int
## 1                                       Alpha                         
## 2                   Logistic Regression Model                         
## 3                                   Intercept    -5.43 [-15.00, -0.60]
## 4                                       Alpha     6.19  [-0.04, 17.70]
## 5  Random-Intercept Multilevel Logistic Model                         
## 31                                  Intercept    -5.43  [-12.64, 1.77]
## 41                                      Alpha     6.19  [-2.73, 15.11]
## 8                                       Omega                         
## 9                   Logistic Regression Model                         
## 51                                  Intercept   -10.30 [-26.37, -0.34]
## 6                                   Omega tot    11.38  [-0.36, 29.55]
## 12 Random-Intercept Multilevel Logistic Model                         
## 7                                   Intercept   -10.30  [-22.35, 1.75]
## 81                                  Omega tot    11.38  [-2.51, 25.27]
##    statistic p.value
## 1                   
## 2                   
## 3      -1.48    .139
## 4       1.36    .174
## 5                   
## 31     -1.48    .139
## 41      1.36    .174
## 8                   
## 9                   
## 51     -1.68    .094
## 6       1.61    .108
## 12                  
## 7      -1.68    .094
## 81      1.61    .108
## 
## term     : Predictor 
## estimate : $b$ 
## conf.int : 95\\% CI 
## statistic: $z$ 
## p.value  : $p$
```

Cronbach’s alpha and omega did not significantly relate to
replication success in the main logistic regression model (Cronbachs
alpha: \(b = 6.19\), 95% CI \([-0.04, 17.70]\), \(z = 1.36\), \(p =
.174\); omega: \(b = 11.38\),
95% CI \([-0.36, 29.55]\), \(z = 1.61\), \(p =
.108\)), nor in any of the multilevel sensitivity analyses models
(see Table @ref(tab:ReplicabilityReliabilityTestTable)). This is
contradictory to the expectation in hypothesis 3. However, the results
from these analyses should be evaluated with caution, given the small
sample number of measures used to estimate these models.

## Hypothesis 4, 5, & 6

Hypothesis 4, 5, & 6 were all tested using a model with the same
dependent variable: total QMP ratio. In order to model a ratio dependent
variable, beta regression was used. Beta regression models operate
similarly to standard generalized linear models with similar
interpretations of the results. What differentiates them is that they
are suitable to model dependent variables with values in the interval of
\((0, 1)\), meaning ratios.
Furthermore, they are well suited for modelling heteroskedastic and
asymmetrically distributed dependent variables [@cribari2010beta]. The beta regression model
was implemented using the betareg function within the betareg package
[@R-betareg\_a; @R-betareg\_b]. Defaults
were used for all function parameters besides the model formula and
data. All beta regression models used the beta link function, which is
as follows:

\[\begin{equation}
g(.) = (0, 1) \mapsto \mathbb{R} (\#eq:BetaLink)
\end{equation}\]

All of these models assume that the QMPs are the same across each
replication set. The reason being that only one protocol was analysed
per replication set, and several of the questions regarding QMPs focus
solely on the reporting of relevant measurement information. This
assumption was deemed likely, since each replication within a set was
based on the same protocol. Furthermore, the goal of these protocols is
to ensure that the measurement practices are equal across the different
replication studies of the same original effect.

## Hypothesis 4

This study seeks to conceptually replicate some of @flake2022construct findings within the Many
Labs replication projects.

- RQ4a. To what degree are QMPs present in replications of
  psychological research?
- RQ4b. To what degree are QMPs present in original psychological
  research?

The structured and preregistered protocols used for the Many Labs
projects were hypothesized to lead to consistent reporting of important
measurement details, and as a result contain less QMPs.

- H4. QMPs are expected to be more frequent in original psychological
  research than in replication research.

### Analysis

To test hypothesis 4, the total QMP ratios between replication and
original research was compared. The model formula for this test looked
as follows:

\[\begin{equation}
g(QMP\\_ratio\_{i}) = \beta\_{0} + \beta\_{1} replication\_{i} (\#eq:H4Main)
\end{equation}\]

Where \(replication\_{i}\) is a dummy
coded variable, which was coded as 1 in case the reported QMP ratio
belonged to a replications, and 0 if it belonged to an original
study.

Results showed that original articles contained a significantly
larger proportion of QMPs than replication protocols for the measures of
the same effects (beta(151) = -0.55, *p* = < .001), and
results based on the initial QMPs showed similar significance (beta(151)
= -0.54, *p* = < .001). This result is in line with hypothesis
4.

## Hypothesis 5

If a replication does not properly measure the variables of the
original effect, the effect analysed within the replication may not be
the same as the original. Establishing a link between QMPs and
non-replicability should confirm suspicions of their detrimental effects
on replications.

- RQ5. What is the association between QMPs in replication studies and
  replication outcome?

Several earlier findings point towards a positive relation between
successful replication and various types of good research practices. A
similar relation is expected for good measurement practices.

- H5. QMPs in replication research are negatively associated with
  replication of an original finding.

### Analysis

For hypothesis 5 a similar formula was used:

\[\begin{equation}
g(QMP\\_ratio\_{i}) = \beta\_{0} + \beta\_{1} replicated\_{i} (\#eq:H5Main)
\end{equation}\]

Where \(replicated\_{i}\) is a dummy
coded variable, which was coded as 1 if the meta-analytic effect in the
replication set matches the original effect in direction at a
significance alpha level of .05, and 0 otherwise. Note here that unlike
in the test for hypothesis 4, \(QMP\\_ratio\_{i}\) refers only to the QMP
ratios of the replications, meaning that original article QMPs were not
included in this analysis.

Results showed that successful replication significantly related to a
decrease in the ratio of QMPs in replication protocols (beta(74) =
-0.51, *p* = .221). This result is in line with hypothesis 5.
However, the results based on the initial QMPs did not show significant
results (beta(74) = -0.45, *p* = .221).

## Hypothesis 6

If essential information on a measure is not available in an original
article, it may be difficult to know the specifics of that measure when
used in a replication. In that case, the lack of proper reporting on
measurement would impact the transparency and validity of future
replications. To provide insight into this issue, an investigation of
the relationship between original and replication QMPs was included.

- RQ6. What is the association between QMPs in original research and
  QMPs in replications of psychological research?

If the relationship exists, it is expected that questionable
measurement practices in an original paper might cause a spill-over
effect of QMPs into the replication. Thus, QMPs are expected to be
associated across original and replication studies.

- H6. Total number of QMPs in original psychological research is
  positively related to total number of QMPs in replication research.

### Analysis

The relationship between original article QMPs and replication
protocol QMPs was modelled to test hypothesis 6. The outcome variable
remains the same as in the model for hypothesis 5. The independent
variable is changed to the QMP ratio for original articles. The
resulting model formula is as follows:

\[\begin{equation}
g(QMP\\_ratio\\_replication\_{i}) = \beta\_{0} + \beta\_{1}
QMP\\_ratio\\_original\_{i} (\#eq:H6Main)
\end{equation}\]

The results showed that total QMP ratio in the original article
significantly predicted total QMP ratio in the subsequent replication
(beta(74) = 1.56, *p* = .005), which provides some evidence in
favor of hypothesis 6. However, this relation was not significant for
the QMP ratios obtained with the initial protocol (beta(75) = -0.14,
*p* = .785).

## Pre-registration Analysis Justification & Reason for Omission

Follow-up analyses were preregistered for hypothesis 6. The goal of
these analyses was to see if there are any type specific carry-over
effects in QMPs between original and replication. Theses analyses were
changed from the pre-registered analyses to beta-regressions for each
unique combination of the QMP categories across original and
replication, as the original analysis using contingency tables was later
deemed improper for our data and hypothesis 6. Furthermore, these models
no longer converged in this version of the article and so are therefore
omitted. These analyses can be found in the first pre-print release of
this project in the supplementary materials.
