# Supplementary material for: Measurement reliability, construct validity, and transparent reporting in original and replication psychological research
Source: PLoS One. 2026 Sep 2;21(9):e0356070. doi: 10.1371/journal.pone.0356070 (PMC13537538; doi:10.1371/journal.pone.0356070)
Supplement: Supplementary file 3 [file pone.0356070.s002.html]

Supplementary\_initial\_QMP\_ratio\_table.knit


```
# Seed for random number generation
set.seed(17042023)
knitr::opts_chunk$set(cache.extra = knitr::rand_seed)
```

# Supplementary Analyses B: Initial Coding Protocol QMP Table

This appendix contains the QMP Table equivalent based on the QMPs
obtained using the initial coding protocol.

### Explanation

The data was initially coded using to the preregistered coding
protocol. After the coding was completed using the preregistered coding
protocol, it was determined that some of the initial ratings were
possibly too stringent. As a result, some of the items in the protocol
were reformulated, so that the conclusions from the resulting analyses
were based on a more lenient judgement of QMPs. The coded data using the
initial protocol was still separately available, and because this
protocol was the one that was pre-registered the QMP table based on
these QMPs is shown here.

### Table

Table @ref(tab:QMPtableInitial) below shows for both original and
replication for all of the QMP items the ratio of QMPs and for how many
of the 77 measures the QMP was applicable. Meanwhile, the Phi
coefficient indicates the “correlation” between the MP being
questionable or good in the original and the same for the replication.
Compared to the revised protocol, the QMP rates are much higher,
especially for modification items as we had specific criteria for this
that required things to be reported all in the article/protocol with
specific details for what should be there.

```
# create informative Measurement Practice Labels 
MPractice <- c("Reliability is reported", "It is clear if the measure existed or is newly created",    "Specifc version of the measure is specified", "Validity evidence from a factor analysis is presented",   "The measured variable is defined", "The choice of measure selection or creation is justified",    "The implemented operationalisation is justified", "The source of the measure is provided",    "Psychometric evidence from the study is given", "Psychometric evidence from an earlier study is given",    "The administration format and environment are described", "The administration procedure is described",   "The number of items are described", "The number of response options are described",    "The recoding of responses is described", "The creation of the index is described",    "Example items are presented in text or supplement", "Any administration format changes are mentioned",    "Administration format changes are justified/validated", "Any translations are mentioned",    "Translated measures are justified/validated", "Any changes in number of items or response options are mentioned",    "Changes in number of items or response options are justified/validated")   


# construct the QMP table to be printed 
QMP_table_initial <- data.frame("Practice" = MPractice, 
                        "QMP original" = paste0(QMP_ratio_data_initial$QMP_percentage_original, " (", QMP_ratio_data_initial$N_applicable_original, ")"), 
                        "QMP Replication" = paste0(QMP_ratio_data_initial$QMP_percentage_replication, " (", QMP_ratio_data_initial$N_applicable_replication, ")"), 
                        "Phi" = QMP_ratio_data_initial$Phi)   


# print the table in apa formatting 
QMP_table_initial
```

```
##                                                                  Practice
## 1                                                 Reliability is reported
## 2                  It is clear if the measure existed or is newly created
## 3                             Specifc version of the measure is specified
## 4                   Validity evidence from a factor analysis is presented
## 5                                        The measured variable is defined
## 6                The choice of measure selection or creation is justified
## 7                         The implemented operationalisation is justified
## 8                                   The source of the measure is provided
## 9                           Psychometric evidence from the study is given
## 10                   Psychometric evidence from an earlier study is given
## 11                The administration format and environment are described
## 12                              The administration procedure is described
## 13                                      The number of items are described
## 14                           The number of response options are described
## 15                                 The recoding of responses is described
## 16                                 The creation of the index is described
## 17                      Example items are presented in text or supplement
## 18                        Any administration format changes are mentioned
## 19                  Administration format changes are justified/validated
## 20                                         Any translations are mentioned
## 21                            Translated measures are justified/validated
## 22       Any changes in number of items or response options are mentioned
## 23 Changes in number of items or response options are justified/validated
##    QMP.original QMP.Replication   Phi
## 1     0.67 (39)       0.89 (37) -0.27
## 2     0.52 (77)       0.96 (77)  -0.5
## 3     0.53 (76)       0.16 (76)  0.39
## 4     0.95 (41)          1 (37) -0.15
## 5     0.21 (70)       0.47 (70) -0.27
## 6     0.52 (77)        0.1 (77)  0.45
## 7      0.4 (77)       0.12 (77)  0.33
## 8     0.57 (76)       0.14 (77)  0.44
## 9     0.92 (38)          1 (36)  -0.2
## 10    0.76 (38)       0.92 (37) -0.21
## 11    0.69 (77)       0.17 (77)  0.52
## 12    0.27 (77)       0.03 (77)  0.35
## 13    0.15 (75)       0.14 (77)  0.01
## 14    0.31 (68)       0.14 (64)   0.2
## 15    0.54 (26)       0.71 (17) -0.17
## 16    0.48 (48)       0.43 (44)  0.05
## 17    0.48 (77)       0.29 (76)   0.2
## 18        1 (6)       0.29 (34)  0.51
## 19        1 (2)        0.8 (25)  0.13
## 20        1 (5)       0.52 (44)  0.29
## 21        1 (1)        0.9 (29)  0.06
## 22     0.67 (6)       0.12 (26)  0.52
## 23     0.67 (3)       0.59 (22)  0.05
```
