# Supplementary material for: Measurement reliability, construct validity, and transparent reporting in original and replication psychological research
Source: PLoS One. 2026 Sep 2;21(9):e0356070. doi: 10.1371/journal.pone.0356070 (PMC13537538; doi:10.1371/journal.pone.0356070)
Supplement: Supplementary file 4 [file pone.0356070.s003.html]

Supplementary\_item\_reduced\_measures\_analyses.knit


```
# Seed for random number generation
set.seed(17042023)
knitr::opts_chunk$set(cache.extra = knitr::rand_seed)
```

```
# combine replication and original data 
is_replication <- c(rep(FALSE, 77), rep(TRUE, 77))

combi_org_rep <- rbind(coded_data_original, coded_data_replications)

combi_org_rep <- cbind(combi_org_rep, is_replication)

combi_org_rep$has_reliability <- combi_org_rep$reliability_type != "Not Reported" & combi_org_rep$reliability_type != ""


# function to calculate QMP ratios
calculating <- function(Data){
  QMP_data <- data.frame(def_ratio = rep(NA, 154),
                         op_ratio = rep(NA, 154),
                         sel_ratio = rep(NA, 154),
                         quant_ratio = rep(NA, 154),
                         mod_ratio = rep(NA, 154),
                         QMP_ratio = rep(NA, 154),
                         op_REV_ratio = rep(NA, 154),
                         sel_REV_ratio = rep(NA, 154),
                         quant_REV_ratio = rep(NA, 154),
                         mod_REV_ratio = rep(NA, 154),
                         QMP_REV_ratio = rep(NA, 154))
  
  def_count <- as.numeric(Data$def_1)
  def_count[is.na(def_count)] <- 0
  def_total <- as.numeric(!is.na(Data$def_1))
  QMP_data$def_ratio <- 1 - round(def_count / def_total, 4) 
  QMP_data$def_ratio[is.na(Data$def_ratio)] <- 0
  
  op_count <- rowSums(Data[16:20], na.rm = TRUE)
  op_total <- rowSums(!is.na(Data[16:20]))
  QMP_data$op_ratio <- 1 - round(op_count / op_total, 4)
  
  sel_count <- rowSums(Data[23:26], na.rm = TRUE)
  sel_total <- rowSums(!is.na(Data[23:26]))
  QMP_data$sel_ratio <- 1 - round(sel_count / sel_total, 4) 
  
  quant_count <- rowSums(Data[29:32], na.rm = TRUE)
  quant_total <- rowSums(!is.na(Data[29:32]))
  QMP_data$quant_ratio <- 1 - round(quant_count / quant_total, 4)  
  
  mod_count <- rowSums(Data[34:39], na.rm = TRUE)
  mod_total <- rowSums(!is.na(Data[34:39]))
  QMP_data$mod_ratio <- 1 - round(mod_count / mod_total, 4) 
  
  MP_total <- def_total + op_total + sel_total + quant_total + mod_total
  MP_count <- def_count + op_count + sel_count + quant_count + mod_count
  QMP_data$QMP_ratio <- 1 - round(MP_count / MP_total, 4) 
  
  op_REV_count <- rowSums(Data[c(18,19,41,42,43)], na.rm = TRUE)
  op_REV_total <- rowSums(!is.na(Data[c(18,19,41,42,43)]))
  QMP_data$op_REV_ratio <- 1 - round(op_REV_count / op_REV_total, 4) 
  
  sel_REV_count <- rowSums(Data[c(24,26,44,45)], na.rm = TRUE)
  sel_REV_total <- rowSums(!is.na(Data[c(24,26,44,45)]))
  QMP_data$sel_REV_ratio <- 1 - round(sel_REV_count / sel_REV_total, 4)  
  
  quant_REV_count <- rowSums(Data[c(32,48,49,50)], na.rm = TRUE)
  quant_REV_total <- rowSums(!is.na(Data[c(32,48,49,50)]))
  QMP_data$quant_REV_ratio <- 1 - round(quant_REV_count / quant_REV_total, 4) 
  
  mod_REV_count <- rowSums(Data[52:57], na.rm = TRUE)
  mod_REV_total <- rowSums(!is.na(Data[52:57]))
  QMP_data$mod_REV_ratio <- 1 - round(mod_REV_count / mod_REV_total, 4) 
  
  MP_REV_count <- def_count + op_REV_count + sel_REV_count + quant_REV_count + mod_REV_count
  MP_REV_total <- def_total + op_REV_total + sel_REV_total + quant_REV_total + mod_REV_total
  QMP_data$QMP_REV_ratio <- 1 - round(MP_REV_count / MP_REV_total, 4) 
  
  return(QMP_data)
}

# calculating the QMP ratios for each QMP item.
QMP_Ratio_data <- calculating(combi_org_rep)

QMP_Ratio_data <- cbind(QMP_Ratio_data, is_replication)
```

# Supplementary Analyses C: Item Reduced Measures Analyses

This appendix contains the analyses on the impact that measures with
a changed number of items had on the main results from Supplementary
Analyses A. These analyses were not pre-registered, but were conducted
to check for the potentially confounding influence of these measures.
The results of these supplementary analyses show that this impact was
minimal overall.

There were overall 7 measures for which the number of items were
reduced. In the following analyses we will see their impact on the
results

```
# getting information on hypothesis support/replication success
hypothesis_support_index <- coded_data_replications$hypothesis_support

hypothesis_support_index[coded_data_replications$hypothesis_support == "Unclear"] <- "Yes"


# chi-squared test of differences in hypothesis support/replication success 
# between measures for which items were decreased and for which it was not.
decreased_items_replicability_impact_test <- chisq.test(decreased_items_index, coded_data_replications$hypothesis_support)
```

```
## Warning in chisq.test(decreased_items_index,
## coded_data_replications$hypothesis_support): Chi-squared approximation may be
## incorrect
```

```
decreased_items_replicability_impact_test_result <- paste0("$Chi^2 = ", apa_num(decreased_items_replicability_impact_test$statistic), "$, df = ",
decreased_items_replicability_impact_test$parameter, "$, p = ",
apa_p(decreased_items_replicability_impact_test$p.value), "$")
```

Interestingly, when item numbers decreased, the replicability seemed
to have increased. Although the difference was not significant (\(Chi^2 = 5.53\), df = 2\(, p = .063\)), and there were too few
modifications to the items to get a stable estimate.

## Reliability

### H1

```
# H1
coded_data_original$reliability_type[decreased_items_index]
```

```
## [1] Not Reported Not Reported Not Reported alpha                    
## [6] Not Reported Not Reported
## Levels:  alpha Not Reported Other retest
```

```
coded_data_replications$reliability_type[decreased_items_index]
```

```
## [1] Not Reported              Not Reported Not Reported             
## [6]              Not Reported
## Levels:  alpha Not Reported retest
```

Only one of these measures had a reported reliability in the
original, so it is unlikely to have been of much impact.

### H2 & H3

```
# H2 & H3
coded_data_original$reliability_coeff[decreased_items_index]
```

```
## [1]   NA   NA   NA 0.62   NA   NA   NA
```

```
coded_data_replications$reliability_coeff[decreased_items_index]
```

```
## [1] NA NA NA NA NA NA NA
```

```
# running the H3 model
measure_reliability_data[measure_reliability_data$reporting_index %in% as.numeric(rownames(item_number_change_data[item_number_change_data$direction == "decrease" & !is.na(item_number_change_data$direction),])),]
```

```
## This is the original data set. The first 6 rows are:
## 
##  [1] alpha                             omega.tot                        
##  [3] omega.hier                        ASE                              
##  [5] tau                               QEp                              
##  [7] pi.lb                             pi.ub                            
##  [9] g                                 coded_data_index                 
## [11] replication_success               reported_coefficient             
## [13] coefficient_difference            significance_reported_coefficient
## <0 rows> (or 0-length row.names)
```

Of the measures in the replication protocol that we could calculate
the reliability of, none from what we can observe in our coded data was
decreased in item count.

## Measurement Reporting

### H4

```
# first we combine the datasets of the original studies and the replications 
# together
# adding "Rep_" to separate replication from original columns in the combined dataset
data_h4_Rep <- QMP_Ratio_data[QMP_Ratio_data$is_replication,]
colnames(data_h4_Rep) <- paste0("Rep_", colnames(data_h4_Rep))

# combine the dataset
data_h4 <- cbind(QMP_Ratio_data[!QMP_Ratio_data$is_replication,], data_h4_Rep)


# comparing the mean and sd original QMP ratio 
print(c("mean decreased" = mean(data_h4$QMP_REV_ratio[decreased_items_index]),
      "sd decreased" = sd(data_h4$QMP_REV_ratio[decreased_items_index]),
      
      "mean non-decreased" = mean(data_h4$QMP_REV_ratio[-decreased_items_index]),
      "sd non-decreased" = sd(data_h4$QMP_REV_ratio[-decreased_items_index])))
```

```
##     mean decreased       sd decreased mean non-decreased   sd non-decreased 
##          0.3397714          0.2398679          0.3089276          0.1782675
```

No clear difference in original QMPs between modified and
non-modified measures. Original measures weren’t modified after all, but
if QMPs were high for the changed measures, the change may be justified
from the standpoint that: change was necessary, the original measurement
had too many QMPs.

```
# comparing the mean and sd replication QMP ratio 
print(c("mean decreased" = mean(data_h4$Rep_QMP_REV_ratio[decreased_items_index]),
      "sd decreased" = sd(data_h4$Rep_QMP_REV_ratio[decreased_items_index]),
      
      "mean non-decreased" = mean(data_h4$Rep_QMP_REV_ratio[-decreased_items_index]),
      "sd non-decreased" = sd(data_h4$Rep_QMP_REV_ratio[-decreased_items_index])))
```

```
##     mean decreased       sd decreased mean non-decreased   sd non-decreased 
##          0.2617286          0.1575185          0.1748474          0.1388186
```

The measures for which the number of items was reduced, had somewhat
more QMPs than those measures for which this was not the case.
Suggesting that when the number of items are reduced, there are likely
also other issues at play.

```
# running the h4 models
# only decreased item measures
betareg(QMP_REV_ratio ~ is_replication, data = QMP_Ratio_data[decreased_items_index,])
```

```
## 
## Call:
## betareg(formula = QMP_REV_ratio ~ is_replication, data = QMP_Ratio_data[decreased_items_index, 
##     ])
## 
## Coefficients (mean model with logit link):
##        (Intercept)  is_replicationTRUE  
##            -0.6754             -0.2938  
## 
## Phi coefficients (precision model with identity link):
## (phi)  
## 5.823
```

```
# only non-decreased item measures
betareg(QMP_REV_ratio ~ is_replication, data = QMP_Ratio_data[!decreased_items_index,])
```

```
## 
## Call:
## betareg(formula = QMP_REV_ratio ~ is_replication, data = QMP_Ratio_data[!decreased_items_index, 
##     ])
## 
## Coefficients (mu model with logit link):
##        (Intercept)  is_replicationTRUE  
##            -0.6325             -0.5648  
## 
## Phi coefficients (phi model with identity link):
## (phi)  
##    13  
## 
## Exceedence parameter (extended-support xbetax model)
## nu: 0.161
```

```
# all (originally)
betareg(QMP_REV_ratio ~ is_replication, data = QMP_Ratio_data)
```

```
## 
## Call:
## betareg(formula = QMP_REV_ratio ~ is_replication, data = QMP_Ratio_data)
## 
## Coefficients (mu model with logit link):
##        (Intercept)  is_replicationTRUE  
##            -0.6389             -0.5454  
## 
## Phi coefficients (phi model with identity link):
## (phi)  
## 11.63  
## 
## Exceedence parameter (extended-support xbetax model)
## nu: 0.1419
```

The effect is in the same direction, but smaller and no longer
significant (although this could also be due to reduction in sample
size). Overall, their removal had almost no impact on the results.

### H5

```
## running the h5 models
# first making data with and without decreased item measures
Diff_QMP_Ratio_data <- QMP_Ratio_data[QMP_Ratio_data$is_replication,]
Diff_QMP_Ratio_data <- Diff_QMP_Ratio_data[decreased_items_index,]
UnDiff_QMP_Ratio_data <- QMP_Ratio_data[QMP_Ratio_data$is_replication,]
UnDiff_QMP_Ratio_data <- UnDiff_QMP_Ratio_data[-decreased_items_index,]
Diff_coded_data_replications <- coded_data_replications[decreased_items_index,]
UnDiff_coded_data_replications <- coded_data_replications[-decreased_items_index,]


# running the model using the QMPs from the revised coding protocol
betareg(Diff_QMP_Ratio_data$QMP_REV_ratio ~ Diff_coded_data_replications$hypothesis_support)
```

```
## 
## Call:
## betareg(formula = Diff_QMP_Ratio_data$QMP_REV_ratio ~ Diff_coded_data_replications$hypothesis_support)
## 
## Coefficients (mean model with logit link):
##                                            (Intercept)  
##                                             -7.968e-17  
## Diff_coded_data_replications$hypothesis_supportUnclear  
##                                             -1.546e+00  
##     Diff_coded_data_replications$hypothesis_supportYes  
##                                             -1.217e+00  
## 
## Phi coefficients (precision model with identity link):
## (phi)  
## 15.18
```

```
betareg(UnDiff_QMP_Ratio_data$QMP_REV_ratio ~ UnDiff_coded_data_replications$hypothesis_support)
```

```
## 
## Call:
## betareg(formula = UnDiff_QMP_Ratio_data$QMP_REV_ratio ~ UnDiff_coded_data_replications$hypothesis_support)
## 
## Coefficients (mu model with logit link):
##                                              (Intercept)  
##                                                  -0.8652  
## UnDiff_coded_data_replications$hypothesis_supportUnclear  
##                                                  -0.5049  
##     UnDiff_coded_data_replications$hypothesis_supportYes  
##                                                  -0.4461  
## 
## Phi coefficients (phi model with identity link):
## (phi)  
## 20.05  
## 
## Exceedence parameter (extended-support xbetax model)
## nu: 0.228
```

```
betareg(QMP_Ratio_data[QMP_Ratio_data$is_replication,]$QMP_REV_ratio ~ coded_data_replications$hypothesis_support)
```

```
## 
## Call:
## betareg(formula = QMP_Ratio_data[QMP_Ratio_data$is_replication, ]$QMP_REV_ratio ~ 
##     coded_data_replications$hypothesis_support)
## 
## Coefficients (mu model with logit link):
##                                       (Intercept)  
##                                           -0.8656  
## coded_data_replications$hypothesis_supportUnclear  
##                                           -0.5082  
##     coded_data_replications$hypothesis_supportYes  
##                                           -0.4400  
## 
## Phi coefficients (phi model with identity link):
## (phi)  
## 20.42  
## 
## Exceedence parameter (extended-support xbetax model)
## nu: 0.2263
```

The association between replication QMP ratio and replicability was
stronger for measures whose items were decreased, but difficult to say
with so few observations. Furthermore, their removal had negligible
impact on the results.

### H6

```
# running the h6 models
betareg(QMP_Ratio_data[!QMP_Ratio_data$is_replication,]$QMP_REV_ratio[decreased_items_index] ~ QMP_Ratio_data[QMP_Ratio_data$is_replication,]$QMP_REV_ratio[decreased_items_index])
```

```
## 
## Call:
## betareg(formula = QMP_Ratio_data[!QMP_Ratio_data$is_replication, ]$QMP_REV_ratio[decreased_items_index] ~ 
##     QMP_Ratio_data[QMP_Ratio_data$is_replication, ]$QMP_REV_ratio[decreased_items_index])
## 
## Coefficients (mean model with logit link):
##                                                                          (Intercept)  
##                                                                              -0.9138  
## QMP_Ratio_data[QMP_Ratio_data$is_replication, ]$QMP_REV_ratio[decreased_items_index]  
##                                                                               1.0446  
## 
## Phi coefficients (precision model with identity link):
## (phi)  
## 4.569
```

```
betareg(QMP_Ratio_data[!QMP_Ratio_data$is_replication,]$QMP_REV_ratio[!decreased_items_index] ~ QMP_Ratio_data[QMP_Ratio_data$is_replication,]$QMP_REV_ratio[!decreased_items_index])
```

```
## 
## Call:
## betareg(formula = QMP_Ratio_data[!QMP_Ratio_data$is_replication, ]$QMP_REV_ratio[!decreased_items_index] ~ 
##     QMP_Ratio_data[QMP_Ratio_data$is_replication, ]$QMP_REV_ratio[!decreased_items_index])
## 
## Coefficients (mu model with logit link):
##                                                                           (Intercept)  
##                                                                               -0.9927  
## QMP_Ratio_data[QMP_Ratio_data$is_replication, ]$QMP_REV_ratio[!decreased_items_index]  
##                                                                                1.6391  
## 
## Phi coefficients (phi model with identity link):
## (phi)  
## 10.47  
## 
## Exceedence parameter (extended-support xbetax model)
## nu: 0.0762
```

```
betareg(QMP_Ratio_data[!QMP_Ratio_data$is_replication,]$QMP_REV_ratio ~ QMP_Ratio_data[QMP_Ratio_data$is_replication,]$QMP_REV_ratio)
```

```
## 
## Call:
## betareg(formula = QMP_Ratio_data[!QMP_Ratio_data$is_replication, ]$QMP_REV_ratio ~ 
##     QMP_Ratio_data[QMP_Ratio_data$is_replication, ]$QMP_REV_ratio)
## 
## Coefficients (mu model with logit link):
##                                                   (Intercept)  
##                                                       -0.9958  
## QMP_Ratio_data[QMP_Ratio_data$is_replication, ]$QMP_REV_ratio  
##                                                        1.5613  
## 
## Phi coefficients (phi model with identity link):
## (phi)  
## 9.177  
## 
## Exceedence parameter (extended-support xbetax model)
## nu: 0.0626
```

The association between original and replication QMPs was reduced and
no longer significant for measures whose items were decreased, but again
there are limited observations for which the number of items were
decreased. Overall, their removal had limited impact on the results.
