# Supplementary material for: Measurement reliability, construct validity, and transparent reporting in original and replication psychological research
Source: PLoS One. 2026 Sep 2;21(9):e0356070. doi: 10.1371/journal.pone.0356070 (PMC13537538; doi:10.1371/journal.pone.0356070)
Supplement: Supplementary file 5 [file pone.0356070.s004.html]

Supplementary\_Omega\_analyses.knit


```
# Seed for random number generation
set.seed(17042023)
knitr::opts_chunk$set(cache.extra = knitr::rand_seed)
```

# Supplementary Analyses D: Omega Results

This appendix contains

We additionally estimated McDonald’s Omega for each lab from the same
set of replications as for Cronbach’s Alpha, since it has been argued to
be a more informative measure of reliability than Cronbach’s Alpha,
while also simultaneously providing some validity evidence [@crutzenScaleQualityAlpha2017b;
@dengTestingDifferenceReliability2017]. McDonald’s Omega was
calculated using the omega function in the *psych* R package
[*v2.6.5*; @R-psych]. Default
arguments were used in the function except the nfactors argument, which
was set to 1. No RG Meta-Analysis was performed on McDonald’s Omega, as
we could not find a formula for calculating its standard error.

## Results

```
is_mostly_na <- function(x){
  return((sum(is.na(x)) / length(x)) > .5) 
}

Omega_convergence_vector <- tapply(calculated_reliability_lab_data$omega.tot, calculated_reliability_lab_data$g, is_mostly_na)

# sum(Omega_convergence_vector)
```

Figure @ref(fig:PlotOmegaDistributions) below shows the result of our
Omega analyses. Note that while we show the distributions of omega
values here, due to the lack of a formula to calculate the Omega
standard error, we did not perform a meta-analysis on these results.
Overall, our Omega coefficient analyses show a similar pattern between
measures to that observed for the alpha coefficient albeit with
generally higher coefficient scores across measures. However, not all
improved. The two columns beside Figure @ref(fig:PlotOmegaDistributions)
show in how many labs each measure was used, as well as for how many
labs an omega coefficient could be obtained, which was not always. In
42.24% of labs, no omega coefficient could be obtained. For 9 measures
the number of labs for which an Omega coefficient could not be obtained
was even the majority. This relates to our findings from the
unidimensionality tests, where some factor models did not converge.

```
# plot for omega
ggplot(calculated_reliability_lab_data, aes(x = omega.tot, y = g)) +
  geom_boxplot(outlier.shape = NA) +
  geom_point(alpha = 0.1) + 
  
  # adding labels to show the number of labs per measure for which a total omega could be calculated
  geom_text(data = calculated_reliability_lab_data[1:19,], label = table(calculated_reliability_lab_data$g[!is.na(calculated_reliability_lab_data$omega.tot)]), x = 1.15, y = 1:19, size = 2.8) +
  # adding labels to show the number of labs per measure
  geom_text(data = calculated_reliability_lab_data[1:19,], label = table(calculated_reliability_lab_data$g), x = 1.35, y = 1:19, size = 2.8) +
  
  # theming
  theme_minimal() +
  theme(legend.position = "none", plot.margin = unit(c(1, 6.5, 1, 1), 
                                                     "lines")) +
  
  # adding the replication line
  geom_hline(yintercept = 6.5, color = "red", size = 1) +
  
  # adding the necessary indicative texts
  annotation_custom(grob = textGrob(label = "Not Replicated", hjust = 0, gp = gpar(fontsize = 10)), ymin = 7.25, ymax = 7.25, xmin = 0.01, xmax = 0.01) +
  annotation_custom(grob = textGrob(label = "Replicated", hjust = 0, gp = gpar(fontsize = 10)), ymin = 6, ymax = 6, xmin = 0.01, xmax = 0.01) +
  annotation_custom(grob = textGrob(label = "N.Omg", hjust = 0, gp = gpar(fontsize = 12)), ymin = 20.2, ymax = 20.2, xmin = 1.08, xmax = 1.08) +
  annotation_custom(grob = textGrob(label = "N.Tot", hjust = 0, gp = gpar(fontsize = 12)), ymin = 20.2, ymax = 20.2, xmin = 1.28, xmax = 1.28) +
  
  coord_cartesian(xlim = c(0, 1), clip = "off") +
  
  ylab("") +
  xlab("Omega")
```

Distributions of calculated McDonald’s Omega t calculated for the
responses on a measure at each lab location, across the eighteen
measures for which raw data was available from which McDonald’s Omega t
coefficients could be calculated. The N.Omg column besides the figure
shows the number of lab locations for which an Omega coefficient could
be successfully calculated. Due to lack of underlying model convergence
an Omega coefficient could not be calculated for all lab locations, or
any lab locations for the Der Fruyt et al. (2000) measure. The N.tot
column shows the total number of lab locations at which the measure was
taken. Any difference between N.omg and N.tot can be seen as an
indication of the instability to get a stable estimate of an underlying
facto model for some lab locations for that measure.
