# Supplementary material for: Measurement reliability, construct validity, and transparent reporting in original and replication psychological research
Source: PLoS One. 2026 Sep 2;21(9):e0356070. doi: 10.1371/journal.pone.0356070 (PMC13537538; doi:10.1371/journal.pone.0356070)
Supplement: Supplementary file 6 [file pone.0356070.s006.docx]

Response to Reviewers

Dear Editor,

We would like to thank both you and the reviewers for the insightful comments. We have clarified the description of the CFA analyses of our manuscript, and revised the exact and LRT fit test descriptions to be accurate and clearer. We also made minor grammatical corrections and improvements throughout the manuscript.

We address all comments point by point below, where our replies to the reviewers are in **bold**, and copied sections from the revised manuscripts are in ***bold italics*** and in quotation marks.

We thank you for considering our manuscript for publication in PLoS One and are looking forward to reading your thoughts.

Yours sincerely, also on behalf of my co-authors,

Cas Goos

## Reviewer #1:

The authors have addressed all the comments intelligently and the manuscript is now clearer and more cohesive, with better synchronized between the aims, results, and discussion. The revisions strengthen the theoretical and practical implications, clarify key concepts, and improve the overall quality and methodological rigor of the study. I thank the authors for their positive efforts indeed.

**We are glad that our revisions were satisfactory to Reviewer 1.**

## Reviewer #2:

Thank you for the opportunity to review the revised manuscript. In my view, the authors have responded constructively to the previous comments, and the manuscript is now substantially improved. The main concerns about overinterpretation, the limited basis for claims about selective or biased reporting, and the psychometric analyses have been largely addressed.

First, the authors should further soften a small number of statements that still sound stronger than the evidence allows. For example, the abstract states that poor reporting “often obscures insufficient reliability and validity.” Given the limited number of measures for which alpha could be recalculated, a more cautious formulation such as “may obscure” or “can obscure” would be preferable. Similar caution should be maintained in the conclusion.

**We thank the reviewer for pointing out these instances were caution in the interpretation of the effects was not yet sufficiently applied.**

**We therefore adjusted the following sections:**

**Abstract Page 2, changed to: *“may obscure insufficient reliability and validity”***

**Discussion Page 34, added: *“based on our tests”* to *“*Some measures showed poor fit, while others showed good fit based on our tests - though not consistently, and fit also depended on invariance constraints imposed across groups and labs.*”***

**Conclusion Page 40, added: *“when reported, our analyses suggest it was often insufficient for some measures across a concerning number of contexts”* to *“when reported, our analyses suggest it was often insufficient for some measures across a concerning number of contexts.”***

Second, the CFA and measurement-invariance reporting should be clarified. The manuscript currently refers to the exact-fit test in a way that could be misleading, as statistical significance in a standard chi-square exact-fit test does not indicate adequate fit.

**We changed the following phrases to more clearly delineate the purpose and use of the exact fit test:**

**Method Section Page 17, rewrote fit analysis description to: *“We also used the exact fit test to test if the model implied covariance matrix for the configurally invariant model matches the data variance covariance matrix tested for measurement invariance across groups against the .05 level. Additionally, we tested with a likelihood ratio test (LRT) if the fit of the weak invariant model significantly differed from the fit of the configurally invariant model, and the fit of the strongly invariant model from the weak at the .05 level.”***

In addition, the interpretation of the invariance tests should be checked carefully. For instance, the statement that “the fit test showed significant measurement invariance for all measures” appears inconsistent with the usual interpretation of significant weak/strong invariance tests, which indicate worsened fit and therefore potential non-invariance. The authors should revise this section to make clear which tests assess global fit, which compare nested invariance models, and how each result should be interpreted.

**Results Section Page 30, changed to: *“For the configurally invariant multi-group models based on the exact fit test, all the unidimensional models rejected the hypothesis that the model implied covariance matrix matches the data variance covariance matrix which is not surprising given our total sample size and number of groups.”***

**Results Section Page 30, changed to: *“The LRT test showed worsened fit due to the invariance restriction of factor loadings for all but one measure”***

**Results Section Page 30, changed to: *“for AIC for all but one measure the fit worsened, and with the added restriction of invariant item intercepts the LRT test indicated that fit for all measures significantly deteriorated.”***

Finally, the manuscript would benefit from a final language and copy-editing pass. There are still minor typographical and phrasing issues, such as “mull model” instead of “null model,” “it we deemed,” and some awkward wording in the conclusion.

**We adjusted numerous small typographical, grammatical, and phrasing issues throughout the text.**
