# Supplementary material for: Measurement reliability, construct validity, and transparent reporting in original and replication psychological research
Source: PLoS One. 2026 Sep 2;21(9):e0356070. doi: 10.1371/journal.pone.0356070 (PMC13537538; doi:10.1371/journal.pone.0356070)
Supplement: Peer review history

# Peer review: Decision Letter 0

**DOI:** 10.1371/journal.pone.0356070.r001

**Authors:** Diego Forero

5 May 2026

PONE-D-25-66287

Measurement Reliability, Construct Validity, and Transparent Reporting in Original and Replication Psychological Research

PLOS One

Dear Dr. Goos,

Thank you for submitting your manuscript to PLOS ONE. After careful consideration, we feel that it has merit but does not fully meet PLOS ONE’s publication criteria as it currently stands. Therefore, we invite you to submit a revised version of the manuscript that addresses the points raised during the review process.

I agree with both reviewers on the need for revisions of the submitted manuscript.

Please submit your revised manuscript by Jun 21 2026 11:59PM. If you will need more time than this to complete your revisions, please reply to this message or contact the journal office at plosone@plos.org. When you're ready to submit your revision, log on to https://www.editorialmanager.com/pone/ and select the 'Submissions Needing Revision' folder to locate your manuscript file.

Please include the following items when submitting your revised manuscript:

- A letter that responds to each point raised by the academic editor and reviewer(s). You should upload this letter as a separate file labeled 'Response to Reviewers'.
- A marked-up copy of your manuscript that highlights changes made to the original version. You should upload this as a separate file labeled 'Revised Manuscript with Track Changes'.
- An unmarked version of your revised paper without tracked changes. You should upload this as a separate file labeled 'Manuscript'.

If you would like to make changes to your financial disclosure, please include your updated statement in your cover letter. Guidelines for resubmitting your figure files are available below the reviewer comments at the end of this letter.

If applicable, we recommend that you deposit your laboratory protocols in protocols.io to enhance the reproducibility of your results. Protocols.io assigns your protocol its own identifier (DOI) so that it can be cited independently in the future. For instructions see: https://journals.plos.org/plosone/s/submission-guidelines#loc-laboratory-protocols. Additionally, PLOS ONE offers an option for publishing peer-reviewed Lab Protocol articles, which describe protocols hosted on protocols.io. Read more information on sharing protocols at https://plos.org/protocols?utm_medium=editorial-email&utm_source=authorletters&utm_campaign=protocols.

As the corresponding author, your ORCID iD is verified in the submission system and will appear in the published article. PLOS supports the use of ORCID, and we encourage all coauthors to register for an ORCID iD and use it as well. Please encourage your coauthors to verify their ORCID iD within the submission system before final acceptance, as unverified ORCID iDs will not appear in the published article. Only the individual author can complete the verification step; PLOS staff cannot verify ORCID iDs on behalf of authors.

We look forward to receiving your revised manuscript.

Kind regards,

Diego A. Forero, MD; PhD

Academic Editor

PLOS One

Journal Requirements:

When submitting your revision, we need you to address these additional requirements.

1. Please ensure that your manuscript meets PLOS ONE's style requirements, including those for file naming. The PLOS ONE style templates can be found at

https://journals.plos.org/plosone/s/file?id=wjVg/PLOSOne_formatting_sample_main_body.pdf and

https://journals.plos.org/plosone/s/file?id=ba62/PLOSOne_formatting_sample_title_authors_affiliations.pdf

2. Please note that PLOS One has specific guidelines on code sharing for submissions in which author-generated code underpins the findings in the manuscript. In these cases, we expect all author-generated code to be made available without restrictions upon publication of the work. Please review our guidelines at https://journals.plos.org/plosone/s/materials-and-software-sharing#loc-sharing-code and ensure that your code is shared in a way that follows best practice and facilitates reproducibility and reuse.

3. If the reviewer comments include a recommendation to cite specific previously published works, please review and evaluate these publications to determine whether they are relevant and should be cited. There is no requirement to cite these works unless the editor has indicated otherwise.

[Note: HTML markup is below. Please do not edit.]

Reviewers' comments:

Reviewer's Responses to Questions

**Comments to the Author**

1. Is the manuscript technically sound, and do the data support the conclusions?

Reviewer #1: Yes

Reviewer #2: Partly

**********

2. Has the statistical analysis been performed appropriately and rigorously?

Reviewer #1: Yes

Reviewer #2: No

**********

3. Have the authors made all data underlying the findings in their manuscript fully available?

The PLOS Data policy

Reviewer #1: Yes

Reviewer #2: Yes

**********

4. Is the manuscript presented in an intelligible fashion and written in standard English?

Reviewer #1: Yes

Reviewer #2: Yes

**********

Reviewer #1: This is an interesting and well-conducted study addressing an important issue in psychological research, namely the transparency and quality of measurement reporting. The focus on both original and replication studies, particularly within the Many Labs framework, provides a strong and relevant basis for evaluating current practices in the field. The use of openly available data to assess reliability and unidimensionality further strengthens the methodological rigor and contributes to the credibility of the study.

The findings are presented nicely and provides valuable insight into persistent shortcomings in reporting reliability and validity. The discussion appropriately situates the results within the broader literature on the replication crisis and research transparency, and the practical recommendations provided are both relevant and useful for improving future research practices.

I am a little concerned about a few areas to improve the readership value of the manuscript. For instance, providing more detail on the criteria used to define “full” measurement reporting would improve transparency. Additionally, a brief elaboration on the implications of unstable reliability across labs for theory development could further strengthen the discussion. Synchronization among the objectives, results, and discussions would be beneficial. Practical implications may be given with clarity within the concluding remarks.

Overall, this is a meaningful contribution to the literature and will be of interest to researchers concerned with methodological rigor and reproducibility in psychological science. I thank the authors for addressing such a relevant agenda of research.

Reviewer #2: Thank you for the opportunity to read and review this paper. I read the manuscript with interest. The paper addresses an important issue in psychological science, namely the transparency of measurement reporting and the stability of reliability and unidimensionality across Many Labs studies. I believe the manuscript has potential, because the topic is relevant and the combination of reporting review and secondary psychometric analyses is valuable. At the same time, I think the paper needs substantial revision before it can be considered for publication.

My first concern is about the reliability analyses. The number of reported Cronbach’s alphas in the original papers appears quite limited, and this makes it difficult to draw strong conclusions about differences between reported, unreported, and estimated reliabilities. This is particularly relevant when, later in the Discussion, you suggest possible bias in measurement reporting. That interpretation is plausible, but the available empirical basis seems rather limited, and I am not sure the current data are sufficient to support such a conclusion strongly. I did not see formal tests, such as moderation-type analyses, that would directly support that claim, although I understand that such tests may themselves be underpowered given the small number of measures. You do acknowledge related limitations, but the point still seems somewhat too strongly promoted in the conclusions.

My second concern is about the unidimensionality analyses. In my view, these analyses are problematic because many labs may have relatively small sample sizes, and fitting CFA models separately within small samples can produce unstable parameter estimates and unreliable fit indices. In such cases, poor fit may reflect sample size limitations rather than genuine measurement problems. You partly acknowledge convergence problems and the possible role of small sample sizes, but I think this concern should receive more weight in the interpretation. Alternative approaches could include multigroup CFA or multilevel CFA models that explicitly account for clustering by lab, rather than fitting separate models at the lab level. More generally, given the low number of studies and variables ultimately available for these analyses, I am not fully convinced that the results are sufficiently informative to support the broader conclusions currently drawn.

Relatedly, I think it would strengthen the paper if you discussed more explicitly whether the current analytic strategy can recover trustworthy results under realistic conditions. A simulation study reflecting a similar scenario, with small per-lab sample sizes, group effects, and questionnaire responses, could be a useful way to test whether, under a favorable validity scenario, the current approach would still produce apparently poor reliability and fit. With small per-lab samples, observed alpha coefficients can vary substantially simply because of sampling error, and fit indices may also perform poorly. There is considerable methodological literature on these issues, so even a more explicit discussion of that literature would already help if a simulation is beyond the scope of the paper.

Overall, I am more convinced by the descriptive part of the manuscript than by the testing part. The descriptive results on reporting standards in original and replication studies are, in my opinion, informative and worth reporting. By contrast, the analyses of lab-level reliability and factorial validity seem more difficult to interpret, and I am therefore not sure that some of the stronger conclusions fully follow from the available evidence. For this reason, I would encourage you either to frame these analyses more cautiously or to strengthen their methodological justification.

As minor comments, I found Figure 2 difficult to read. The green lines are almost invisible, and the figure does not report the actual sample sizes or even sample size ranges for the lab-level analyses. Adding clearer visual elements and explicit information on the sample sizes would make the figure easier to interpret.

The manuscript would also benefit from some language editing. A few specific examples are: “discrimant” should be corrected to “discriminant” in the Abstract (p. 2, line 27); on p. 3, lines 47–50, the sentence beginning “Psychometrics offers a wide range of tools...” is too long and difficult to follow, and “asses” should be corrected to “assess”. The sentence also finishes without the dot; on p. 8, line 168, “practical wat” should be “practical way”; on p. 17, lines 383–385, the sentence beginning “which was appreciably lower than found in Flake et al. (6). on reliability coefficient reporting...” should be revised for grammar and capitalization; and on p. 14, lines 312–315, the sentence beginning “Our analyses will focus on Cronbach’s Alpha...” contains phrasing that is currently unclear, especially “the standard errors of Alpha caused in the RG meta-analysis.” .

In sum, I think this is an interesting and potentially useful study. However, I believe the descriptive contribution is stronger than the psychometric testing component in its current form. I would therefore encourage a more cautious interpretation of the reliability and unidimensionality analyses, and a clearer alignment between the evidence presented and the conclusions drawn.

**********

what does this mean?). If published, this will include your full peer review and any attached files.

If you choose “no”, your identity will remain anonymous but your review may still be made public.

**Do you want your identity to be public for this peer review?**  For information about this choice, including consent withdrawal, please see our Privacy Policy

Reviewer #1: No

Reviewer #2: **Yes:**  Tommaso Feraco

**********

[NOTE: If reviewer comments were submitted as an attachment file, they will be attached to this email and accessible via the submission site. Please log into your account, locate the manuscript record, and check for the action link "View Attachments". If this link does not appear, there are no attachment files.]

To ensure your figures meet our technical requirements, please review our figure guidelines: https://journals.plos.org/plosone/s/figures

You may also use PLOS’s free figure tool, NAAS, to help you prepare publication quality figures: https://journals.plos.org/plosone/s/figures#loc-tools-for-figure-preparation.

NAAS will assess whether your figures meet our technical requirements by comparing each figure against our figure specifications.

# Author comment: Author response to Decision Letter 1

**DOI:** 10.1371/journal.pone.0356070.r002

20 Jun 2026

Response to Reviewers

Dear Editor,

We would like to thank both you and the reviewers for the insightful comments. We have revised our manuscript ,to improve its clarity and the nuance in our interpretation of its results.

Specifically, We also changed our unidimensionality tests to be more appropriately suited to the data and revised the interpretation of our results to be more cautious and placed them more clearly within the larger context of the literature.

We address all comments point by point below, where our replies to the reviewers are in bold, and copied sections from the revised manuscripts are in bold italics and in quotation marks.

We thank you for considering our manuscript for publication in PLoS One and are looking forward to reading your thoughts.

Your sincerely, also on behalf of my co-authors,

Cas Goos

Reviewer #1:

This is an interesting and well-conducted study addressing an important issue in psychological research, namely the transparency and quality of measurement reporting. The focus on both original and replication studies, particularly within the Many Labs framework, provides a strong and relevant basis for evaluating current practices in the field. The use of openly available data to assess reliability and unidimensionality further strengthens the methodological rigor and contributes to the credibility of the study.

The findings are presented nicely and provides valuable insight into persistent shortcomings in reporting reliability and validity. The discussion appropriately situates the results within the broader literature on the replication crisis and research transparency, and the practical recommendations provided are both relevant and useful for improving future research practices.

REPLY: We would like to thank the reviewer for their positive evaluation of our manuscript, and have sought to ensure that these elements have retained their central place in our manuscript.

I am a little concerned about a few areas to improve the readership value of the manuscript. For instance, providing more detail on the criteria used to define “full” measurement reporting would improve transparency.

REPLY: We agree that defining this more explicitly would improve clarity. We have changed the relevant sections as follows:

Page 12 added: “When all criteria are coded as “true”, we consider the article’s reporting to be “full”, in that the necessary information for both evaluating and reconstructing the measure is reported.”

Page 25 added: “[...] many studies did not fully report sufficient information to enable other researchers to reconstruct the measure”

This way, the term “full” is directly related to how we measured and operationalized the reporting of measurement information.

Additionally, a brief elaboration on the implications of unstable reliability across labs for theory development could further strengthen the discussion.

REPLY: We have changed the relevant sections as follows:

Added the following sentence to Page 5: “As a result, critically testing theories on substantial phenomena is stilted because we cannot establish that the relevant constructs were assessed (14–16).”

Added the following sentence to Page 7: “This may lead researchers to believe that a measure that was reliable in a previous sample will also be reliable on their own, whereas it may very well not be. This can result in incorrect substantive inferences, hampering theory development.”

Added the following sentence to Page 32: “Otherwise, the results of the replication cannot be confidently linked to a psychological phenomenon, undermining its contribution to theory testing (14,69).”

Synchronization among the objectives, results, and discussions would be beneficial.

REPLY: To better synchronize these elements, we have aimed to increase the cohesion by adding the following:

Added On Page 8 the following explicit description of the goals: “The combined goal of our three research aims is to first provide a descriptive account of the measurement in the included Many Labs study pairs based on the reported information. We add to this our own assessment of reliability and unidimensionality to provide an additional source of evidence on the validity that is not influenced by the reporting practices of our sample.”

On Pages 24-25, we added the following to more clearly link the Discussion to this new formulation of the goals: “Finally, recalculated Cronbach’s Alpha and unidimensionality indices showed variability across measures and contexts. This further highlights the importance of transparent reporting on measurement, as the reliability and unidimensionality of a measure in other contexts is not a guarantee that the measurement in any context. Thus, researchers should make sure to assess reliability and unidimensionality in their study as well.”

Finally, the reformulation of the unidimensionality analyses and their interpretation (detailed below) as discussed on Pages 27 is also relevant for aligning our goals, results, and discussion more closely.

Practical implications may be given with clarity within the concluding remarks.

REPLY: We thank the reviewer for bringing to our attention that the practical implications needed to be highlighted in the conclusion. We therefore changed the final sentence of the conclusion (Page 33) as follows: “Fortunately, even small improvements in more widespread adoption of measurement reporting guidelines, data and materials sharing, valid measurement as a prerequisite to substantive interpretation and inclusion in replication projects, especially for single item measures, can spark the proliferation of validated measurement.”

Overall, this is a meaningful contribution to the literature and will be of interest to researchers concerned with methodological rigor and reproducibility in psychological science. I thank the authors for addressing such a relevant agenda of research.

Reviewer #2:

Thank you for the opportunity to read and review this paper. I read the manuscript with interest. The paper addresses an important issue in psychological science, namely the transparency of measurement reporting and the stability of reliability and unidimensionality across Many Labs studies. I believe the manuscript has potential, because the topic is relevant and the combination of reporting review and secondary psychometric analyses is valuable. At the same time, I think the paper needs substantial revision before it can be considered for publication.

My first concern is about the reliability analyses. The number of reported Cronbach’s alphas in the original papers appears quite limited, and this makes it difficult to draw strong conclusions about differences between reported, unreported, and estimated reliabilities. This is particularly relevant when, later in the Discussion, you suggest possible bias in measurement reporting. That interpretation is plausible, but the available empirical basis seems rather limited, and I am not sure the current data are sufficient to support such a conclusion strongly. I did not see formal tests, such as moderation-type analyses, that would directly support that claim, although I understand that such tests may themselves be underpowered given the small number of measures. You do acknowledge related limitations, but the point still seems somewhat too strongly promoted in the conclusions.

REPLY: We agree with the reviewer that upon reinspection our conclusions were still too strongly worded in our discussion section, we therefore rewrote the discussion section on biased reporting to be more tentative and relate the conclusions more to existing literature, and request further research:

Revised on Pages 27-28 : “Previous research has shown an excess of reported Alphas around the commonly acceptable threshold of .70 (11), demonstrating potentially biased reporting in Cronbach’s Alpha coefficients. In line with this, we did observe for our measures that if an original study reported a Cronbach’s Alpha, the corresponding replications typically showed relatively high reliability. Meanwhile, if no Cronbach’s Alpha was reported in the original study, the reliability in the replications was generally lower than if a Cronbach’s Alpha was reported. However, while this narrative would explain why we may observe a lack of reliability and validity reporting, our observation of biased reporting is based on only a small set of measures (19) for which we could calculate the Cronbach’s Alphas, and is therefore too insubstantial to state this claim with certainty. We do, however, consider it to be a candidate to explain at least part of the underreporting observed by us and others, and encourage future research to extend the investigation to a more suitable sample.”

My second concern is about the unidimensionality analyses. In my view, these analyses are problematic because many labs may have relatively small sample sizes, and fitting CFA models separately within small samples can produce unstable parameter estimates and unreliable fit indices. In such cases, poor fit may reflect sample size limitations rather than genuine measurement problems. You partly acknowledge convergence problems and the possible role of small sample sizes, but I think this concern should receive more weight in the interpretation. Alternative approaches could include multigroup CFA or multilevel CFA models that explicitly account for clustering by lab, rather than fitting separate models at the lab level.

REPLY: We thank the reviewer for their suggestion to use multigroup CFA as a more fitting method for our sample and analysis goal. It had not yet occurred to us to use this type of model, and we agree it is more suitable for our data.

A brief description of the model from our manuscript added on Page 14 is as follows: “To assess unidimensionality, we fit a multi-group single-factor model on the item responses from measures with suitable data – meaning at least 3 items that are approximately normally distributed – grouped by lab and any conditions or demographics that were used in the test of the effect in the Many Labs replications. For example, if the measure were used in ten labs and there were two experimental conditions, we would have twenty groups in our comparison.”

More generally, given the low number of studies and variables ultimately available for these analyses, I am not fully convinced that the results are sufficiently informative to support the broader conclusions currently drawn.

REPLY: We again agree that more tentative conclusions are warranted. We have therefore also elaborated on the conclusions based on these analyses to be more clearly tentative.

The most extensive revisions related to this point are on Page 27 (although the tone has been adjusted throughout the article): “We cannot currently state with certainty the degree to which any of these have resulted in the findings we observe. Still, our item response analysis in combination with existing literature indicate that measures are often not assessed on their validity and regularly fail basic psychometric requirements for valid use.

We observed that most measures passed some but not all our unidimensionality checks and that there was considerable variability in reliability among labs for measures with low average reliability across labs too. Some measures showed poor fit, while other measures showed good fit, albeit not consistently and abiding by invariance constraints across different groups and labs . While sufficient validity and measurement invariance is reachable, it is not a universally guaranteed quality of the measures in our sample. Shaw et al. (5) in their analyses of the Many Labs 2 came to similar conclusions regarding the widely ranging reliabilities and inconsistent unidimensional fit. This could mean that if any of the individual labs in the Many Labs projects checked the validity and reliability of their measure before analyzing their results, they might have concluded that their measurement failed to accurately capture the construct of interest, thereby complicating the substantive conclusions. Similar issues in measurement invariance have been noted by Maassen et al. (1).”

Relatedly, I think it would strengthen the paper if you discussed more explicitly whether the current analytic strategy can recover trustworthy results under realistic conditions. A simulation study reflecting a similar scenario, with small per-lab sample sizes, group effects, and questionnaire responses, could be a useful way to test whether, under a favorable validity scenario, the current approach would still produce apparently poor reliability and fit. With small per-lab samples, observed alpha coefficients can vary substantially simply because of sampling error, and fit indices may also perform poorly. There is considerable methodological literature on these issues, so even a more explicit discussion of that literature would already help if a simulation is beyond the scope of the paper.

REPLY: Because it is outside of the scope of the study, we have not done performed the simulation study as was suggested. Instead, we address the reviewer’s point in the following ways:

We added the following caveat to make the power issues explicit in the Discussion section on Pages 26-27 : “Unfortunately, given the complexity of validating a measure, it is difficult to say with certainty which scenario we are in. This is beyond the scope of this article and inconsistent with our analytical approach. Furthermore, the Many Labs projects did not specify their samples to be powered and representative for complete measurement validation. The low per lab sample size and few items — common in the replication measures — add sampling error to our reliability coefficient estimates and would bias the estimates of fit for our unidimensional factor analysis models downward. Furthermore, for the reliability assessment, even under MI and valid measurement, just because of differences in the true factor between groups we would expect different reliabilities across groups. We cannot currently state with certainty the degree to which any of these have resulted in the findings we observe. Still, our item response analysis in combination with existing literature indicate that measures are often not assessed on their validity and regularly fail basic psychometric requirements for valid use.”

We conducted a power analysis similar to Maassen et al. (2015) on our ability to detect measurement invariance in our data, as reported on Page 14 of the manuscript: “For all the included results the power to detect Measurement non-Invariance in the intercept in a third of the items for half of the groups was at least .80, this prerequisite was based on (1).”

Overall, I am more convinced by the descriptive part of the manuscript than by the testing part. The descriptive results on reporting standards in original and replication studies are, in my opinion, informative and worth reporting. By contrast, the analyses of lab-level reliability and factorial validity seem more difficult to interpret, and I am therefore not sure that some of the stronger conclusions fully follow from the available evidence. For this reason, I would encourage you either to frame these analyses more cautiously or to strengthen their methodological justification.

REPLY: We thank the reviewer for their constructive criticism of our article, and we hope that our changes mentioned above will have addressed their concerns sufficiently.

As minor comments, I found Figure 2 difficult to read. The green lines are almost invisible, and the figure does not report the actual sample sizes or even sample size ranges for the lab-level analyses. Adding clearer visual elements and explicit information on the sample sizes would make the figure easier to interpret.

We increased the size and changed the color of the green lines. We also added the Mean sample size per lab as well as the 25th and 75th quantiles of the sample size.

The manuscript would also benefit from some language editing. A few specific examples are: “discrimant” should be corrected to “discriminant” in the Abstract (p. 2, line 27); on p. 3, lines 47–50, the sentence beginning “Psychometrics offers a wide range of

Attachment Submitted filename: Response to Reviewers.docx

# Peer review: Decision Letter 1

**DOI:** 10.1371/journal.pone.0356070.r003

**Authors:** Diego Forero

9 Jul 2026

Dear Dr. Goos,

**I agree with the reviewer on the need for further minor revisions.**

Please submit your revised manuscript by Aug 23 2026 11:59PM. If you will need more time than this to complete your revisions, please reply to this message or contact the journal office at plosone@plos.org. When you're ready to submit your revision, log on to https://www.editorialmanager.com/pone/ and select the 'Submissions Needing Revision' folder to locate your manuscript file.

- A letter that responds to each point raised by the academic editor and reviewer(s). You should upload this letter as a separate file labeled 'Response to Reviewers'.
- A marked-up copy of your manuscript that highlights changes made to the original version. You should upload this as a separate file labeled 'Revised Manuscript with Track Changes'.
- An unmarked version of your revised paper without tracked changes. You should upload this as a separate file labeled 'Manuscript'.

If you would like to make changes to your financial disclosure, please include your updated statement in your cover letter. Guidelines for resubmitting your figure files are available below the reviewer comments at the end of this letter.

If applicable, we recommend that you deposit your laboratory protocols in protocols.io to enhance the reproducibility of your results. Protocols.io assigns your protocol its own identifier (DOI) so that it can be cited independently in the future. For instructions see: https://journals.plos.org/plosone/s/submission-guidelines#loc-laboratory-protocols. Additionally, PLOS ONE offers an option for publishing peer-reviewed Lab Protocol articles, which describe protocols hosted on protocols.io. Read more information on sharing protocols at https://plos.org/protocols?utm_medium=editorial-email&utm_source=authorletters&utm_campaign=protocols.

As the corresponding author, your ORCID iD is verified in the submission system and will appear in the published article. PLOS supports the use of ORCID, and we encourage all coauthors to register for an ORCID iD and use it as well. Please encourage your coauthors to verify their ORCID iD within the submission system before final acceptance, as unverified ORCID iDs will not appear in the published article. *Only*  the individual author can complete the verification step; PLOS staff *cannot*  verify ORCID iDs on behalf of authors.

We look forward to receiving your revised manuscript.

Kind regards,

Diego A. Forero, MD; PhD

Academic Editor

PLOS One

**Journal Requirements:**

1. If the reviewer comments include a recommendation to cite specific previously published works, please review and evaluate these publications to determine whether they are relevant and should be cited. There is no requirement to cite these works unless the editor has indicated otherwise.

2. Please review your reference list to ensure that it is complete and correct. If you have cited papers that have been retracted, please include the rationale for doing so in the manuscript text, or remove these references and replace them with relevant current references. Any changes to the reference list should be mentioned in the rebuttal letter that accompanies your revised manuscript. If you need to cite a retracted article, indicate the article’s retracted status in the References list and also include a citation and full reference for the retraction notice.

[Note: HTML markup is below. Please do not edit.]

Reviewers' comments:

Reviewer's Responses to Questions

**Comments to the Author**

Reviewer #1: (No Response)

Reviewer #2: All comments have been addressed

**********

2. Is the manuscript technically sound, and do the data support the conclusions?

Reviewer #1: Yes

Reviewer #2: Yes

**********

3. Has the statistical analysis been performed appropriately and rigorously?

Reviewer #1: Yes

Reviewer #2: Yes

**********

4. Have the authors made all data underlying the findings in their manuscript fully available?

The PLOS Data policy

Reviewer #1: Yes

Reviewer #2: Yes

**********

5. Is the manuscript presented in an intelligible fashion and written in standard English?

Reviewer #1: Yes

Reviewer #2: Yes

**********

**Reviewer #1:**  The authors have addressed all the comments intelligently and the manuscript is now clearer and more cohesive, with better synchronized between the aims, results, and discussion. The revisions strengthen the theoretical and practical implications, clarify key concepts, and improve the overall quality and methodological rigor of the study. I thank the authors for their positive efforts indeed.

**Reviewer #2:**  Thank you for the opportunity to review the revised manuscript. In my view, the authors have responded constructively to the previous comments, and the manuscript is now substantially improved.

The main concerns about overinterpretation, the limited basis for claims about selective or biased reporting, and the psychometric analyses have been largely addressed.

There are only a few remaining points.

First, the authors should further soften a small number of statements that still sound stronger than the evidence allows. For example, the abstract states that poor reporting “often obscures insufficient reliability and validity.” Given the limited number of measures for which alpha could be recalculated, a more cautious formulation such as “may obscure” or “can obscure” would be preferable. Similar caution should be maintained in the conclusion.

Second, the CFA and measurement-invariance reporting should be clarified. The manuscript currently refers to the exact-fit test in a way that could be misleading, as statistical significance in a standard chi-square exact-fit test does not indicate adequate fit. In addition, the interpretation of the invariance tests should be checked carefully. For instance, the statement that “the fit test showed significant measurement invariance for all measures” appears inconsistent with the usual interpretation of significant weak/strong invariance tests, which indicate worsened fit and therefore potential non-invariance. The authors should revise this section to make clear which tests assess global fit, which compare nested invariance models, and how each result should be interpreted.

Finally, the manuscript would benefit from a final language and copy-editing pass. There are still minor typographical and phrasing issues, such as “mull model” instead of “null model,” “it we deemed,” and some awkward wording in the conclusion.

**********

what does this mean?). If published, this will include your full peer review and any attached files.

If you choose “no”, your identity will remain anonymous but your review may still be made public.

**Do you want your identity to be public for this peer review?**  For information about this choice, including consent withdrawal, please see our Privacy Policy

Reviewer #1: No

Reviewer #2: **Yes:**  Tommaso Feraco

**********

[NOTE: If reviewer comments were submitted as an attachment file, they will be attached to this email and accessible via the submission site. Please log into your account, locate the manuscript record, and check for the action link "View Attachments". If this link does not appear, there are no attachment files.]

To ensure your figures meet our technical requirements, please review our figure guidelines: https://journals.plos.org/plosone/s/figures

You may also use PLOS’s free figure tool, NAAS, to help you prepare publication quality figures: https://journals.plos.org/plosone/s/figures#loc-tools-for-figure-preparation.

NAAS will assess whether your figures meet our technical requirements by comparing each figure against our figure specifications.

# Author comment: Author response to Decision Letter 2

**DOI:** 10.1371/journal.pone.0356070.r004

23 Jul 2026

Dear Editor,

We would like to thank both you and the reviewers for the insightful comments. We have clarified the description of the CFA analyses of our manuscript, and revised the exact and LRT fit test descriptions to be accurate and clearer. We also made minor grammatical corrections and improvements throughout the manuscript.

We thank you for considering our manuscript for publication in PLoS One and are looking forward to reading your thoughts.

Yours sincerely, also on behalf of my co-authors,

Cas Goos

Reviewer #1:

The authors have addressed all the comments intelligently and the manuscript is now clearer and more cohesive, with better synchronized between the aims, results, and discussion. The revisions strengthen the theoretical and practical implications, clarify key concepts, and improve the overall quality and methodological rigor of the study. I thank the authors for their positive efforts indeed.

We are glad that our revisions were satisfactory to Reviewer 1.

Reviewer #2:

Thank you for the opportunity to review the revised manuscript. In my view, the authors have responded constructively to the previous comments, and the manuscript is now substantially improved. The main concerns about overinterpretation, the limited basis for claims about selective or biased reporting, and the psychometric analyses have been largely addressed.

First, the authors should further soften a small number of statements that still sound stronger than the evidence allows. For example, the abstract states that poor reporting “often obscures insufficient reliability and validity.” Given the limited number of measures for which alpha could be recalculated, a more cautious formulation such as “may obscure” or “can obscure” would be preferable. Similar caution should be maintained in the conclusion.

We thank the reviewer for pointing out these instances were caution in the interpretation of the effects was not yet sufficiently applied.

We therefore adjusted the following sections:

Abstract Page 2, changed to: “may obscure insufficient reliability and validity”

Discussion Page 34, added: “based on our tests” to “Some measures showed poor fit, while others showed good fit based on our tests - though not consistently, and fit also depended on invariance constraints imposed across groups and labs.”

Conclusion Page 40, added: “when reported, our analyses suggest it was often insufficient for some measures across a concerning number of contexts” to “when reported, our analyses suggest it was often insufficient for some measures across a concerning number of contexts.”

Second, the CFA and measurement-invariance reporting should be clarified. The manuscript currently refers to the exact-fit test in a way that could be misleading, as statistical significance in a standard chi-square exact-fit test does not indicate adequate fit.

We changed the following phrases to more clearly delineate the purpose and use of the exact fit test:

Method Section Page 17, rewrote fit analysis description to: “We also used the exact fit test to test if the model implied covariance matrix for the configurally invariant model matches the data variance covariance matrix tested for measurement invariance across groups against the .05 level. Additionally, we tested with a likelihood ratio test (LRT) if the fit of the weak invariant model significantly differed from the fit of the configurally invariant model, and the fit of the strongly invariant model from the weak at the .05 level.”

In addition, the interpretation of the invariance tests should be checked carefully. For instance, the statement that “the fit test showed significant measurement invariance for all measures” appears inconsistent with the usual interpretation of significant weak/strong invariance tests, which indicate worsened fit and therefore potential non-invariance. The authors should revise this section to make clear which tests assess global fit, which compare nested invariance models, and how each result should be interpreted.

Results Section Page 30, changed to: “For the configurally invariant multi-group models based on the exact fit test, all the unidimensional models rejected the hypothesis that the model implied covariance matrix matches the data variance covariance matrix which is not surprising given our total sample size and number of groups.”

Results Section Page 30, changed to: “The LRT test showed worsened fit due to the invariance restriction of factor loadings for all but one measure”

Results Section Page 30, changed to: “for AIC for all but one measure the fit worsened, and with the added restriction of invariant item intercepts the LRT test indicated that fit for all measures significantly deteriorated.”

Finally, the manuscript would benefit from a final language and copy-editing pass. There are still minor typographical and phrasing issues, such as “mull model” instead of “null model,” “it we deemed,” and some awkward wording in the conclusion.

We adjusted numerous small typographical, grammatical, and phrasing issues throughout the text.

Attachment Submitted filename: Response to Reviewers (second round).docx

# Peer review: Decision Letter 2

**DOI:** 10.1371/journal.pone.0356070.r005

**Authors:** Diego Forero

30 Jul 2026

Measurement Reliability, Construct Validity, and Transparent Reporting in Original and Replication Psychological Research

PONE-D-25-66287R2

Dear Dr. Goos,

We’re pleased to inform you that your manuscript has been judged scientifically suitable for publication and will be formally accepted for publication once it meets all outstanding technical requirements.

Within one week, you’ll receive an e-mail detailing the required amendments. When these have been addressed, you’ll receive a formal acceptance letter and your manuscript will be scheduled for publication.

An invoice will be generated when your article is formally accepted. Please note, if your institution has a publishing partnership with PLOS and your article meets the relevant criteria, all or part of your publication costs will be covered. Please make sure your user information is up-to-date by logging into Editorial Manager at Editorial Manager® and clicking the ‘Update My Information' link at the top of the page. For questions related to billing, please contact billing support.

If your institution or institutions have a press office, please notify them about your upcoming paper to help maximize its impact. If they’ll be preparing press materials, please inform our press team as soon as possible -- no later than 48 hours after receiving the formal acceptance. Your manuscript will remain under strict press embargo until 2 pm Eastern Time on the date of publication. For more information, please contact onepress@plos.org.

Kind regards,

Diego A. Forero, MD; PhD

Academic Editor

PLOS One

Additional Editor Comments (optional):

Reviewers' comments:

Reviewer's Responses to Questions

**Comments to the Author**

Reviewer #2: All comments have been addressed

**********

2. Is the manuscript technically sound, and do the data support the conclusions?

Reviewer #2: Yes

**********

3. Has the statistical analysis been performed appropriately and rigorously?

Reviewer #2: Yes

**********

4. Have the authors made all data underlying the findings in their manuscript fully available?

The PLOS Data policy

Reviewer #2: Yes

**********

5. Is the manuscript presented in an intelligible fashion and written in standard English?

Reviewer #2: Yes

**********

Reviewer #2: The authors have adequately addressed the main issues raised in my previous review, including the interpretation of the findings, the reporting of the CFA and measurement-invariance analyses, and the remaining language and copy-editing concerns. I have no further comments.

**********

what does this mean?). If published, this will include your full peer review and any attached files.

If you choose “no”, your identity will remain anonymous but your review may still be made public.

**Do you want your identity to be public for this peer review?**  For information about this choice, including consent withdrawal, please see our Privacy Policy

Reviewer #2: **Yes:**  Tommaso Feraco

**********

# Editor report: Acceptance letter

**DOI:** 10.1371/journal.pone.0356070.r006

**Authors:** Diego Forero

PONE-D-25-66287R2

PLOS One

Dear Dr. Goos,

I'm pleased to inform you that your manuscript has been deemed suitable for publication in PLOS One. Congratulations! Your manuscript is now being handed over to our production team.

At this stage, our production department will prepare your paper for publication. This includes ensuring the following:

* All references, tables, and figures are properly cited

* All relevant supporting information is included in the manuscript submission,

* There are no issues that prevent the paper from being properly typeset

You will receive further instructions from the production team, including instructions on how to review your proof when it is ready. Please keep in mind that we are working through a large volume of accepted articles, so please give us a few days to review your paper and let you know the next and final steps.

Lastly, if your institution or institutions have a press office, please let them know about your upcoming paper now to help maximize its impact. If they'll be preparing press materials, please inform our press team within the next 48 hours. Your manuscript will remain under strict press embargo until 2 pm Eastern Time on the date of publication. For more information, please contact onepress@plos.org.

You will receive an invoice from PLOS for your publication fee after your manuscript has reached the completed accept phase. If you receive an email requesting payment before acceptance or for any other service, this may be a phishing scheme. Learn how to identify phishing emails and protect your accounts at https://explore.plos.org/phishing.

If we can help with anything else, please email us at customercare@plos.org.

Thank you for submitting your work to PLOS ONE and supporting open access.

Kind regards,

PLOS ONE Editorial Office Staff

on behalf of

Dr. Diego A. Forero

Academic Editor

PLOS One
